# Transient Ruddlesden–Popper-Type Defects and Their Influence on Grain Growth and Properties of Lithium Lanthanum Titanate Solid Electrolyte

**DOI:** 10.1021/acsnano.4c00706

**Published:** 2024-04-09

**Authors:** Petruša Borštnar, Goran Dražić, Martin Šala, Che-an Lin, Shih-kang Lin, Matjaž Spreitzer, Nina Daneu

**Affiliations:** † Advanced Materials Department, Jožef Stefan Institute, Jamova cesta 39, 1000 Ljubljana, Slovenia; ‡ Jožef Stefan International Postgraduate School, Jamova cesta 39, 1000 Ljubljana, Slovenia; § Department of Materials Chemistry, National Institute of Chemistry, Hajdrihova 19, 1000 Ljubljana, Slovenia; ∥ Department of Analytical Chemistry, National Institute of Chemistry, Hajdrihova 19, 1000 Ljubljana, Slovenia; ⊥ Department of Materials Science and Engineering, National Cheng Kung University, Tainan 70101, Taiwan; # Hierarchical Green-Energy Materials (Hi-GEM) Research Center, National Cheng Kung University, Tainan 70101, Taiwan; ∇ Program on Smart and Sustainable Manufacturing, Academy of Innovative Semiconductor and Sustainable Manufacturing, National Cheng Kung University, Tainan 70101, Taiwan; ⊗ Core Facility Center, National Cheng Kung University, Tainan 70101, Taiwan

**Keywords:** LLTO, solid electrolyte, exaggerated growth, abnormal growth, Li_2_La_2_Ti_3_O_10_, recrystallization

## Abstract

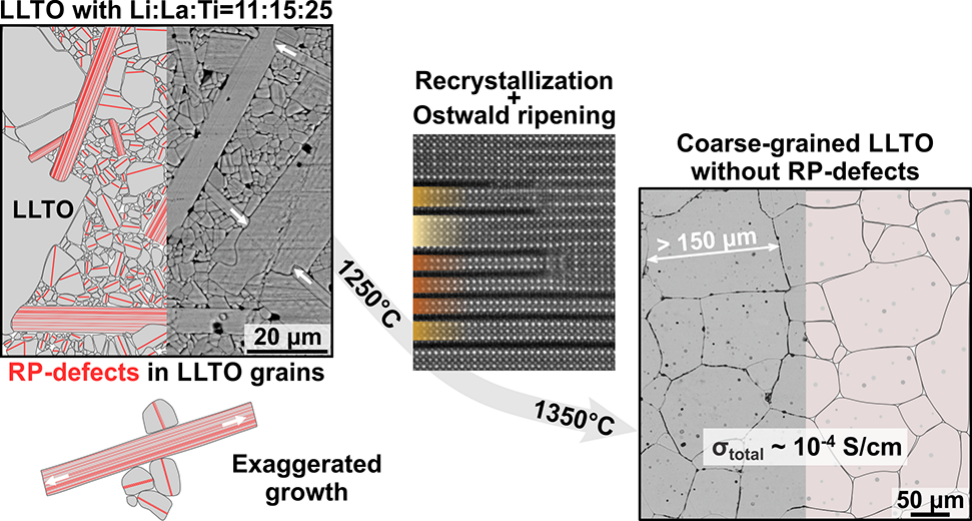

Lithium lanthanum titanate (LLTO) perovskite is one of the most promising electrolytes for all-solid-state batteries, but its performance is limited by the presence of grain boundaries (GBs). The fraction of GBs can be significantly reduced by the preparation of coarse-grained LLTO ceramics. In this work, we describe an alternative approach to the fabrication of ceramics with large LLTO grains based on self-seeded grain growth. In compositions with the starting stoichiometry for the Li_0.20_La_0.60_TiO_3_ phase and with a high excess addition of Li (Li:La:Ti = 11:15:25), microstructure development starts with the formation of the layered RP-type Li_2_La_2_Ti_3_O_10_ phase. Grains with many RP-type defects initially develop into large platelets with thicknesses of up to 10 μm and lengths over 100 μm. Microstructure development continues with the crystallization of LLTO perovskite, epitaxially on the platelets and as smaller grains with thinner in-grain RP-lamellae. Theoretical calculations confirmed that the formation of RP-type sequences is energetically favored and precedes the formation of the LLTO perovskite phase. At around 1250 °C, the RP-type sequences become thermally unstable and gradually recrystallize to LLTO via the ionic exchange between the Li-rich RP-layers and the neighboring Ti and La layers as shown by quantitative HAADF-STEM. At higher sintering temperatures, LLTO grains become free of RP-type defects and the small grains recrystallize onto the large platelike seed grains via Ostwald ripening. The final microstructure is coarse-grained LLTO with total ionic conductivity in the range of 1 × 10^–4^ S/cm.

Increasing energy consumption calls for the development of all-solid-state batteries (ASSB) with high energy and power densities, capacity, stability, and durability.^1,2^ Many studies have focused on finding an inorganic solid electrolyte for the new generation of batteries that would lead to achieving comparable or even improved characteristics as in batteries with liquid electrolytes. It is also important that the materials are environmentally friendly and do not decompose to toxic products.^3^ Compounds from the family of oxide perovskites based on lithium lanthanum titanate Li_3*x*_La_2/3–*x*_□_1/3–2*x*_TiO_3_ (LLTO) are auspicious candidates for solid electrolytes as they exhibit high stability and high bulk ionic conductivity at room temperature.^4,5^

The total ionic conductivity of polycrystalline LLTO-based ceramics is the sum of the grain (bulk) and grain boundary (GB) conductivities. Compositions with *x* between 0.04 and 0.11 can reach grain ionic conductivities up to 10^–3^ S/cm.^5−7^ These phases accommodate La, Li, and vacancies on the A-sites,^8^ which enables fast Li migration through the crystal lattice.^9−12^ The ionic conductivity of LLTO mainly depends on composition (*x*-value),^5,11,13^ sintering regime (cooling rate),^8,9,14^ crystal orientation,^7,15^ and modification of the perovskite phase.^9,11^ In pseudocubic α-LLTO modification, the A-site cations are randomly distributed, and the Li ions can freely migrate through the lattice in a 3D manner. In tetragonal β-LLTO with alternating La-rich and La-poor layers, the ionic transport is slowed because the migration paths for the Li ions are restricted to the La-poor (Li-rich) layers. Tetragonal β-LLTO preferentially forms during slow cooling, while the pseudocubic α-LLTO modification can be stabilized by quenching from temperatures above 1250 °C,^16−19^ which is the β to α-LLTO transition temperature.^8^

While the ionic conductivity of LLTO grains is already suitable for practical applications, the total ionic conductivity of LLTO ceramics is lowered by the presence of 2D and 3D defects such as grain boundaries, domain boundaries, planar defects, secondary phases, and pores, which block the transport of Li ions and decrease the total ionic conductivity by orders of magnitude.^20−26^ Strategies to decrease the contribution of these structural discontinuities to the total ionic conductivity involve processing in a moisture-free environment to avoid the formation of Li-rich secondary phases^27^ and preparation of microstructures with large pseudocubic domains^15^ or LLTO grains with a reduced fraction of GBs.^22,28,29^

We have recently reported an alternative approach to producing coarse-grained LLTO-based ceramics based on triggering abnormal growth of LLTO grains due to the formation of Ruddlesden–Popper (RP)-type planar defects inside LLTO grains.^30^ Abundant nucleation of RP-type defects was observed in compositions with the initial La:Ti ratio of 0.605 and excess addition of Li_2_CO_3_. The formation of the defects caused fast and anisotropic growth of a few selected grains in the initial phase of microstructure development, and these grains act as seeds for the development of coarse-grained ceramics. The final microstructure after sintering at 1350 °C is composed of large LLTO grains without planar defects and the total ionic conductivity of the ceramics was in the range of 10^–4^ S/cm. While the basic principles of microstructure development have been described, the details related to the transient nature of RP-type defects remain unclear. In this study, we used quantitative high-angle annular dark field scanning transmission electron microscopy (HAADF-STEM) in combination with theoretical calculations to obtain deeper insight into the reasons for the early formation and later recrystallization of the RP-type sequences and to understand their influence on the grain growth and functional properties of the LLTO-based solid electrolyte.

## Results and Discussion

### General Microstructural Characteristics

**Figure 1:**
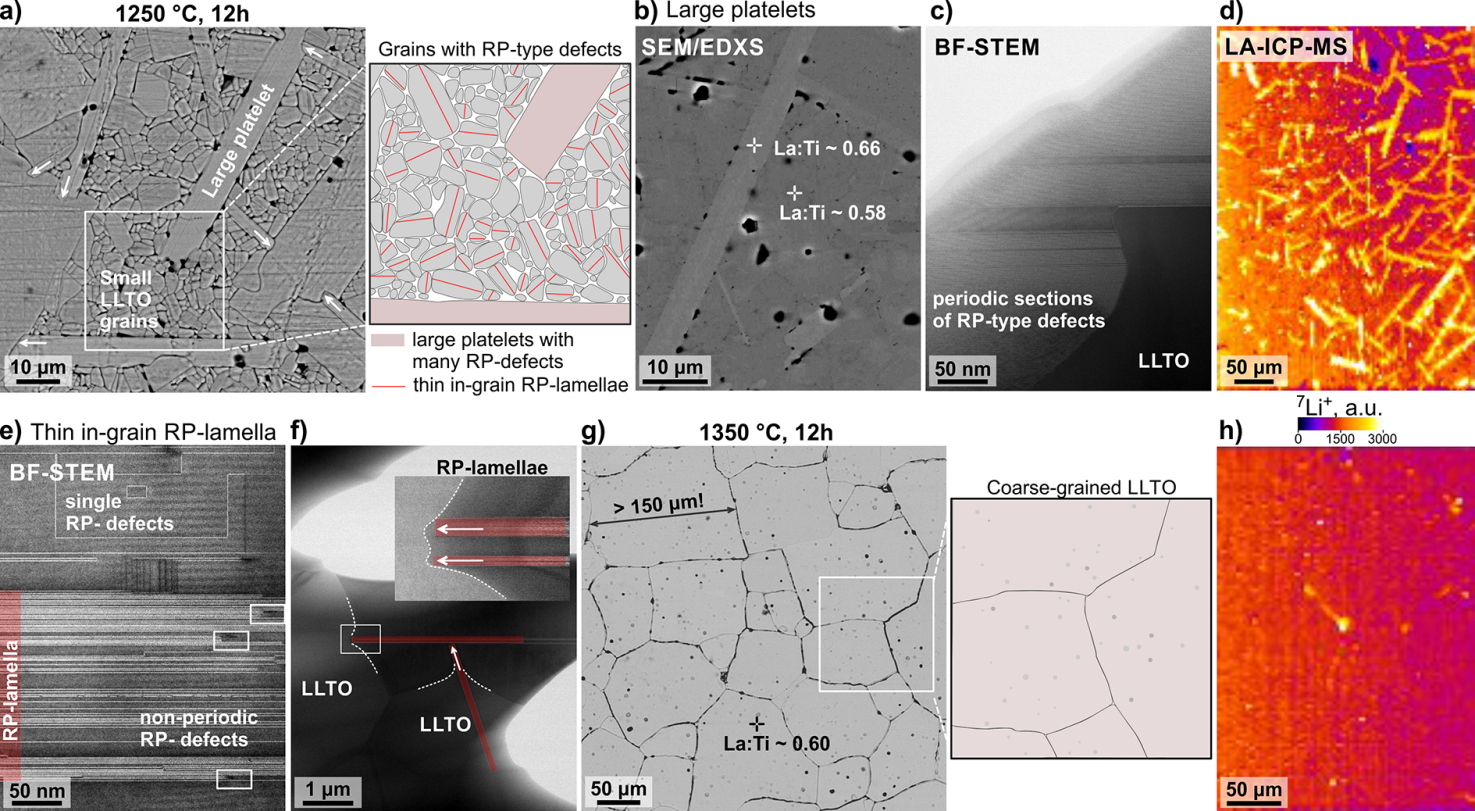
(a) Thermally etched microstructure of the sample with a Li:La:Ti ratio of 11:15:25 after sintering at 1250 °C. The sample is composed of exaggeratedly grown platelets surrounded by small LLTO grains. Arrows indicate the fast growth direction of the platelets. Most of the smaller LLTO grains contain planar defects as indicated in the scheme. (b) In unetched backscattered electron (BSE) images, the platelets are slightly brighter and, according to SEM/EDXS, have a higher average La:Ti ratio than the grains with darker contrast. (c) Bright-field (BF) STEM image of a thicker platelet with many parallel defects which sometimes order into periodic sequences. (d) LA-ICP-MS map of the sample after sintering at 1250 °C shows that the large platelets are enriched in Li. (e) Thin lamella with nonperiodically arranged defects inside a smaller LLTO grain. Recrystallization of the defects to the matrix perovskite (framed areas) can be observed in the samples sintered at 1250 °C. (f) Also the thin in-grain RP-lamellae exhibit faster growth than the matrix LLTO perovskite and sometimes even penetrate the adjacent LLTO grains. (g) After sintering at 1350 °C, most of the LLTO grains are large with La:Ti ratio of around 0.6, and contain many intragranular pores, while RP-type defects inside the grains are no longer observed. (h) According to the LA-ICP-MS map, Li is homogeneously distributed to all grains after sintering at 1350 °C. LA-ICP-MS maps of Li, La, and Ti for samples sintered at 1250 and 1350 °C are shown in Figure S2.

The thermally etched microstructure of the ceramics with a starting Li:La:Ti ratio of 11:15:25 after sintering at 1250 °C is shown in Figure 1a. The sample contains large anisotropic platelike grains with thicknesses up to around 10 μm and lengths exceeding 100 μm, surrounded by smaller grains with more isometric morphology measuring below 10 μm in diameter, most of them are intersected by planar defects. According to powder XRD (Figure S1), the sample contains only tetragonal LLTO, and the amount of secondary phases is below the detection limit. The formation of tetragonal LLTO is favored due to the low sintering temperature of 1250 °C in combination with slow cooling.^8,19^ The large platelets exhibit characteristics of preferential and exaggerated growth^31,32^ and sometimes intrude other large grains (situations marked by arrows in Figure 1a). According to SEM/EDXS, they have a La:Ti ratio of around 0.66 and exhibit a slightly brighter contrast in backscattered electron (BSE) images (Figure 1b) in comparison to the surrounding smaller grains with a lower La:Ti ratio of around 0.58 that corresponds to the LLTO perovskite with an *x*-value around 0.09. As revealed by BF-STEM, the large platelets are densely populated with parallel defects (Figure 1c). As discussed in our previous study^30^ these sequences are planar defects with structural elements of the RP-type Li_2_La_2_Ti_3_O_10_ phase. According to the stoichiometry, the Li_2_La_2_Ti_3_O_10_ phase contains about 2.3 wt % of Li, whereas the LLTO phases contain a lower fraction of Li, for example, the LLTO (*x* = 0.09) contains only around 1.1 wt % of Li. Laser ablation inductively coupled plasma mass spectrometry (LA-ICP-MS) was used to confirm that the large platelike grains are enriched in Li (Figure 1d). In contrast to the large platelets, the smaller LLTO grains contain planar defects in the form of thinner in-grain lamellae composed of nonperiodically arranged parallel defects (Figure 1e).

After sintering at 1250 °C, recrystallization of RP-type defects to LLTO can be observed; a few situations are indicated in Figure 1e. Similar to the large platelets, the in-grain lamellae exhibit characteristics of exaggerated grain growth. Typically, they grow faster than their matrix LLTO grains and sometimes even penetrate deeply into the neighboring grains (Figure 1f). The matrix LLTO grains next to the in-grain lamellae occasionally contain single defects along the equivalent {100} directions, which can form closed loops (Figure S3). These defects have the highest density right next to the in-grain lamellae as observed in the upper part of Figure 1e, and they appear as single-atom-layer traps (SALTs) described by Zhu et al. (2020).^33^ After sintering at 1350 °C, the ceramic is composed of large LLTO grains with average grain size 110 μm and with some grains measuring more than 250 μm in diameter. According to SEM/EDXS, average La:Ti ratio of the grains is around 0.6, which is close to the ratio in the starting powder (Figure S4), and the presence of RP-type lamellae inside the LLTO grains is no longer observed (Figure 1g). Large grains contain many intragranular pores, which form due to the fast grain growth^30^ and partially due to Li evaporation during high-temperature sintering. The LA-ICP-MS map of this sample reveals that Li is homogeneously distributed in the LLTO grains with few indications of Li enrichment at the GBs (Figure 1h). Decreasing content of Li with increasing temperature treatment was confirmed by inductively coupled plasma optical emission spectrometry (ICP-OES) and the results are shown in Table S1.

### Periodic RP-Type Li_2_La_2_Ti_3_O_10_ Sequences

**Figure 2:**
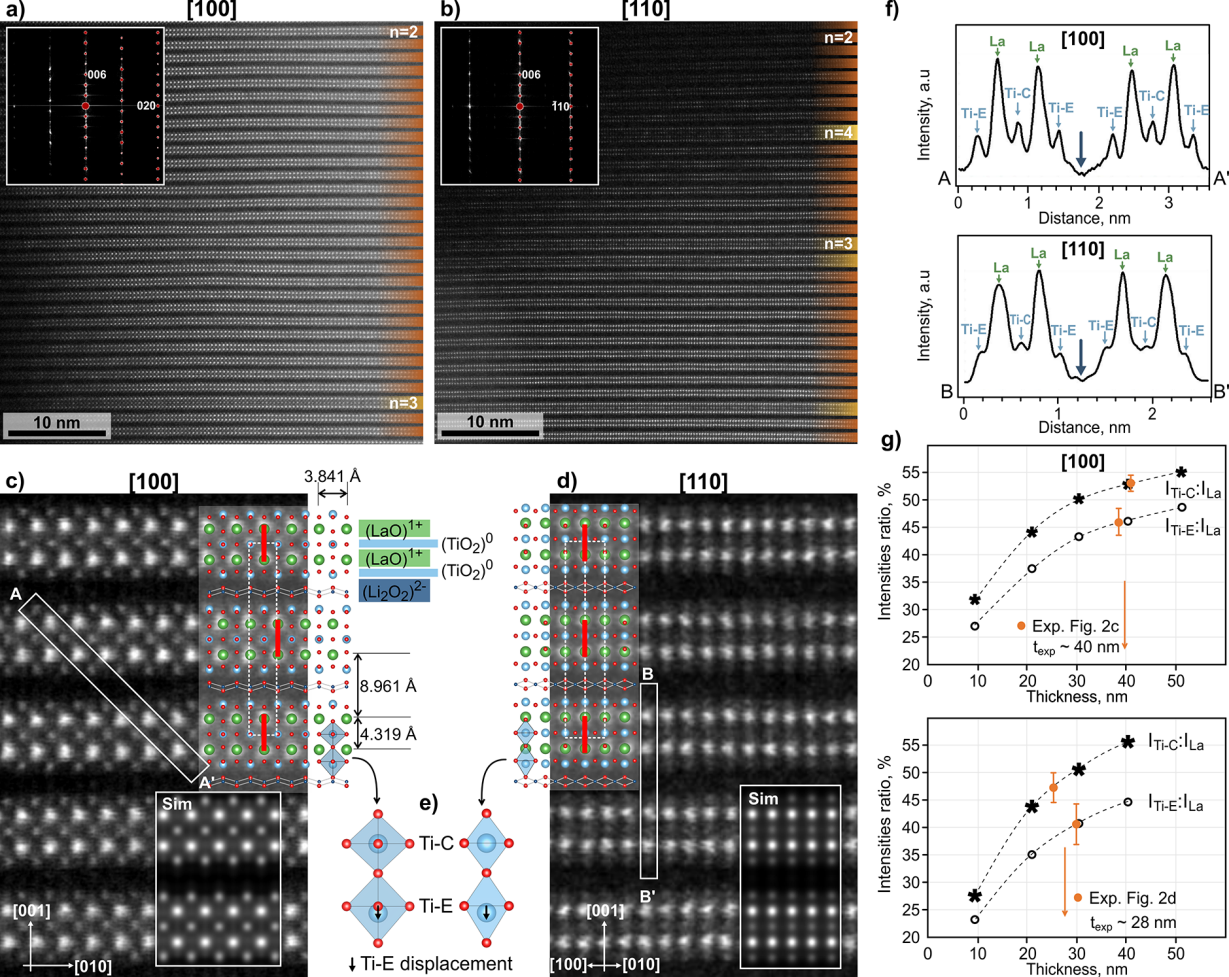
HAADF-STEM images of the periodic Li_2_La_2_Ti_3_O_10_ phase (*n* = 2) with the corresponding FFT patterns along (a) [100] and (b) [110] zone axes. (c,d) High-resolution HAADF-STEM images with superimposed Li_2_La_2_Ti_3_O_10_ structural model. (e) Central and displaced positions of the Ti–C and Ti–E cations inside the two different types of octahedral layers of the pseudoperovskite blocks. (f) A–A′ and B–B′ intensity profiles marked on (c) and (d) show different intensities of Ti–C and Ti–E columns. (g) *I*_Ti–C_:*I*_La_ and *I*_Ti–E_:*I*_La_ intensity ratios with the sample thickness calculated from image simulations. The values determined from experimental images (c) and (d) are marked with orange and yield thicknesses of around 40 and 28 nm, respectively. Image simulations (Sim) for the calculated sample thicknesses are overlaid on HAADF-STEM images in parts (c) and (d).

The larger platelets with many parallel RP-type defects (Figure 1b,c) occasionally contain periodic Li_2_La_2_Ti_3_O_10_ sequences with thicknesses of up to a few tens of nanometers. High-resolution HAADF-STEM images of the Li_2_La_2_Ti_3_O_10_ phase oriented along the [100] and [110] zone axes and the corresponding FFT patterns with superimposed calculated diffraction patterns for each zone axis are shown in Figures 2a,b. The images disclose the layered nature of Li_2_La_2_Ti_3_O_10_ along the *c*-axis, composed of two subsequent La-rich layers (*n* = 2) with bright contrast separated by Li-rich layers with dark contrast. It can be observed that even the largely periodic sequences are occasionally interrupted by perovskite blocks with *n* > 2.

The main structural features of Li_2_La_2_Ti_3_O_10_ along the [100] and [110] zone axes are presented in higher-magnification HAADF-STEM images with superimposed structural models (Figure 2c,d). The pseudoperovskite blocks are composed of two La–O layers that interchange with octahedral Ti–O_2_ layers. It is important to emphasize that in Li_2_La_2_Ti_3_O_10_, all available 12-fold coordinated interstices inside the La–O layers are occupied with La atoms (100% occupancy), whereas in LLTO perovskites, the occupancy of these sites with La is up to 67% (theoretically in La_0.67_TiO_3_) and depends on the composition (the Li content) and symmetry of the phase. The average charge of the La–O layers is 1+ (La^3+^–O^2–^), while the Ti–O_2_ layers are electroneutral (Ti^4+^–2·O^2–^), yielding overall electropositive (La_2_Ti_3_O_8_)^2+^ pseudoperovskite blocks. The subsequent blocks are separated by electronegative (Li_2_–O_2_)^2–^ layers (2·Li^+^–2·O^2–^), which compensate for the positive charge of the pseudoperovskite blocks. The (100) layers of the pseudoperovskite blocks are shifted for one-half of the unit cell in the [010] direction across the Li-rich layer (marked by red lines in Figure 2c), whereas there is no shift in the [110] zone axis (Figure 2d). The shift and the presence of two interlayer cations per formula unit are characteristic for Ruddlesden–Popper (RP) phases.^34,35^ A distinctly layered structure of Li_2_La_2_Ti_3_O_10_ resembles the structure of Bi_4_Ti_3_O_12_ Aurivillius phase, where (Bi_2_O_2_)^2+^ layers separate the (Bi_2_Ti_3_O_10_)^2–^ perovskite blocks.^36^

The described characteristics of the Li_2_La_2_Ti_3_O_10_ phase in terms of the average charge of the atomic layers along the c-direction (out-of-plane) are reflected in the average spacings between the atomic layers in different directions. Overall, the periodic Li_2_La_2_Ti_3_O_10_ sequences have tetragonal symmetry with out-of-plane spacings between the La–O layers within the pseudoperovskite blocks of 4.319 Å, whereas the spacings between the La–O layers across the Li_2_–O_2_ layer are more than twice the value, i.e., 8.961 Å. The distance between the (010) layers (in-plane) is 3.841 Å (Figure 2c). Due to the interchanging of atomic layers with different compositions and average charges, the Ti–O_2_ octahedral layers are of two types; in the central Ti–O_2_ layers (marked as Ti–C) positioned symmetrically between two La–O layers of the pseudoperovskite block, the Ti atoms are centered in the Ti–O_6_ octahedra, whereas in the edge Ti–O_2_ layers (Ti–E), next to the Li_2_–O_2_ layers, the Ti cations are slightly displaced toward the Li-rich layer (Figure 2e). In experimental HAADF-STEM images, the Ti–E columns have slightly lower intensity in comparison to the Ti–C columns, as shown on the A–A′ and B–B′ intensity profiles in [100] and [110] zone axes (Figure 2f). In the [100] zone axis, the Ti-columns in the Ti–E layers do not coincide with the O-positions as in the Ti–C layer, which could be the reason for the lower intensity of the Ti–E columns in Z-contrast images. However, in the [110] projection, where the Ti-column positions are separated from the O-column positions (Figure 2e), the projected composition of Ti–C and Ti–E is identical, but the intensity of the Ti–E columns in HAADF-STEM images is still lower in comparison to the Ti–C columns. We compared experimental images to image simulations to confirm that the lower intensity of the Ti–E atomic columns stems from the structural features and not the lower occupancy. We determined intensity ratios (*I*_Ti–E_:*I*_La_ and *I*_Ti–C_:*I*_La_) for simulated samples with different thicknesses since the atomic column intensities are a function of sample thickness.^37,38^ The results given in Table S2 and graphically presented in Figure 2g show that the lower intensity of the Ti–E columns relative to the Ti–C columns is well reproduced in simulations for both projections, confirming that the absolute intensity of an atomic column in HAADF-STEM depends not only on the average Z of the atomic column but also on its local neighborhood. Baladés et al. (2019) have shown that the intensity of an atomic column with identical composition changes in dependence on the distance and composition of the neighboring atomic columns. For example, an atomic column surrounded by columns with higher average Z (as Ti–C) will have higher intensity due to the transfer of probe intensity from neighboring columns, also referred to as crosstalk.^39,40^ Thicknesses of the sample areas shown in Figures 2c,d of 40 and 28 nm were obtained by comparison of the experimentally determined intensity ratios with values obtained for simulations at different thicknesses (Figure 2g). Simulations for both orientations for the determined sample thicknesses are overlaid on the experimental images (Figure 2c,d), and their intensity profiles (for comparison with the experimental profiles in Figure 2f) across two pseudoperovskite blocks are shown in Figure S5. In addition to the different intensities of the Ti–C and Ti–E columns, it is also important to highlight that no significant increase of the intensity inside the Li_2_–O_2_ layers was detected (marked with arrows in the profiles in Figure 2f). This is expected since, in Li_2_La_2_Ti_3_O_10_, only Li and O atoms reside in this layer and are too light to yield contrast in HAADF-STEM. The high amount of Li in the thicker platelets with dense sequences of the Li_2_La_2_Ti_3_O_10_ phase was confirmed by LA-ICP-MS (Figure 1d).

### Sequences with Nonperiodically Arranged Parallel RP-Type Defects

**Figure 3:**
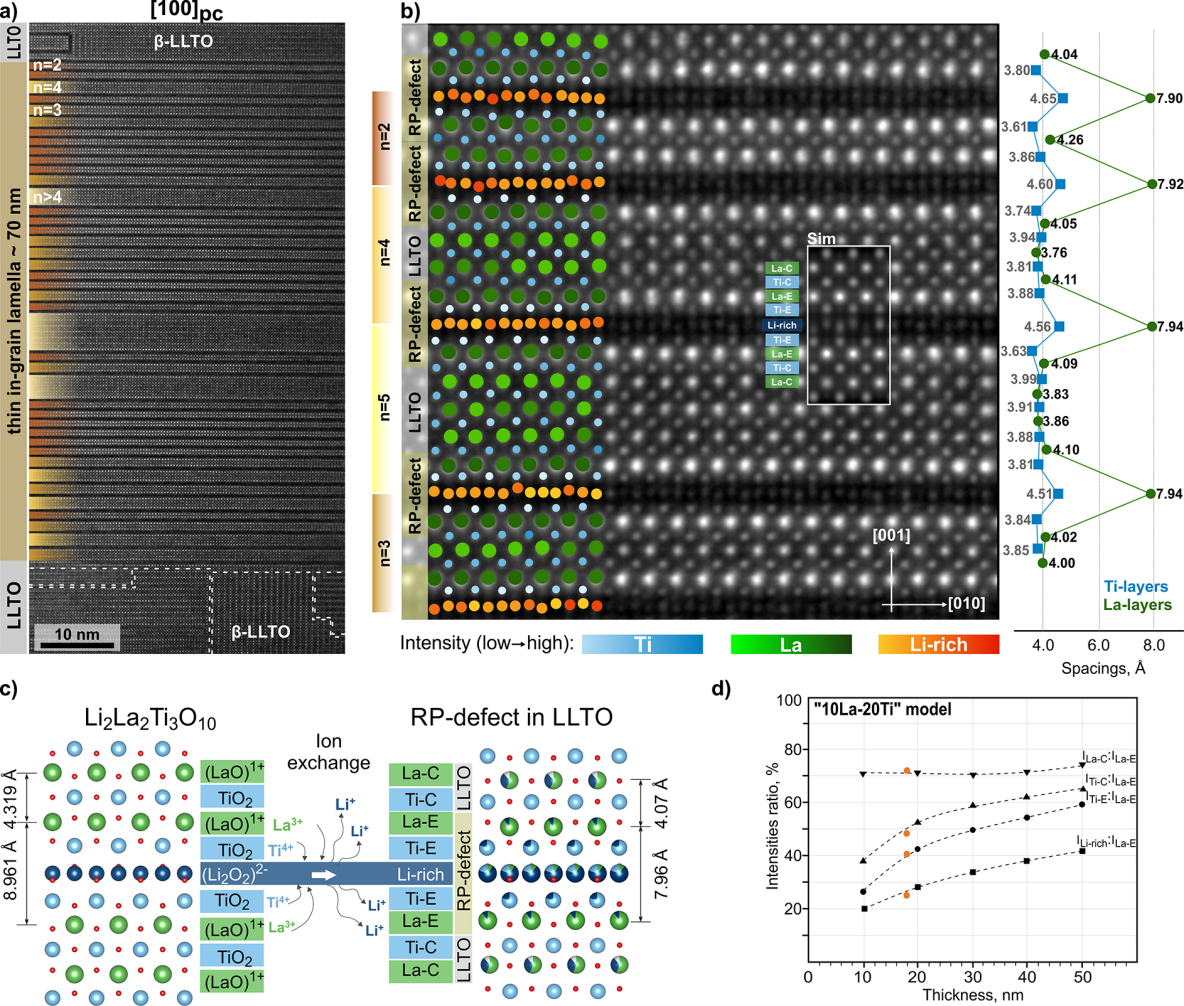
(a) In-grain lamella with nonperiodic structure along the [100]_pc_ zone axes, where Li-rich layers separate pseudoperovskite blocks with variable thicknesses (*n*). (b) Higher magnification image of the nonperiodic sequence with overlaid intensities of the La, Ti, and Li-rich atomic columns (left). The measured distances between the subsequent La- and Li-rich layers (green) and Ti-layers (blue) in the [001] direction are shown on the right. (c) The starting model of an RP-type defect is based on the Li_2_La_2_Ti_3_O_10_ structure (left). Ion exchange between the Li-rich layer and the neighboring La–E and Ti–E layers (right) results in relaxations of the atomic layers around the RP-defect. (d) Intensity ratios for the “10La-20Ti” model at different thicknesses calculated from image simulations. Experimental values fit best at a thickness of around 18 nm (without an amorphous layer).

All lamellae with RP-type defects, the thicker ones that constitute the large platelets as well as the thinner ones inside the smaller LLTO grains, are much more commonly composed of nonperiodically arranged Li-rich layers that separate perovskite blocks with variable thicknesses, i.e., a different number of La-rich layers (*n*). Approximately 70 nm thin in-grain lamella with nonperiodic structure is shown in Figure 3a. The thinnest perovskite blocks comprise two La-rich layers (*n* = 2) and correspond to segments of the Li_2_La_2_Ti_3_O_10_ phase. The thicker LLTO blocks (*n* > 2) have additional LLTO perovskite layers sandwiched between the Li-rich layers. Subsequent perovskite blocks are RP-shifted. The LLTO inside the perovskite blocks with *n* > 4 resembles α-LLTO with a disordered arrangement of the A-site ions, whereas the matrix LLTO outside the lamella has a domain structure composed of a few tens of nanometers large tetragonal β-LLTO domains with interchanging La-rich and La-poor atomic layers. β-LLTO perovskite is the main phase of this sample as confirmed by XRD (Figure S1).

Structural characteristics of the nonperiodic sequences in terms of (heavy) atomic column positions and their intensities were studied in more detail in the high-resolution HAADF-STEM image shown in Figure 3b. The image is a stack of 10 frames acquired at a higher scanning speed (dwell time of 4 μs/px, image size 512 × 512 px) to minimize image distortions due to specimen drift and scanning distortions and improve the signal-to-noise ratio. We used 2D Gaussian fitting (“Gauss fit on spot” plugin, ImageJ) for the determination of atomic column positions with subpixel accuracy. The overall thickness of the area of 29.6 nm was determined by EELS mapping (Figure S6).

It can be observed that La layers of the nonperiodic lamella have different average intensities. The La layers of the RP-defect layers, which are also edge layers of the perovskite blocks (La–E layers), have the highest intensity (*I*_La–E_ = 4769 ± 6%). The La–E layers correspond to the La-layers in Li_2_La_2_Ti_3_O_10_, but here, in the nonperiodic sequences, they exhibit a higher deviation of intensities indicating that their occupancy is lower than 100%. The La columns inside the perovskite blocks with *n* > 2, the La–C columns, have about 72% intensity relative to the La–E columns and an even higher standard deviation of intensities (*I*_La–C_ = 3448 ± 10%). This indicates lower and random occupancy of La–C columns with La as expected for pseudocubic Li_3*x*_La_2/3–*x*_□_1/3–2*x*_TiO_3_ with *x* around 0.11. Another significant difference between the periodic and nonperiodic sequences is higher intensity inside the Li-rich layers of the RP-type defects (the (Li_2_O_2_)^2–^ layers in Li_2_La_2_Ti_3_O_10_), suggesting the presence of heavier atoms. In Figure 3b, the average intensity of the atomic columns in the Li-rich layer is about 25% relative to the intensity of the La–E layers (*I*_Li-rich_ = 1200 ± 12%). We also measured absolute intensities of the atomic columns in the octahedral Ti–O_2_ layers and observed a similar trend as in the periodic Li_2_La_2_Ti_3_O_10_ sequences; the intensity of the Ti–E columns is lower (*I*_Ti–E_ = 1945 ± 6%) than the intensity of the Ti–C columns (*I*_Ti–C_ = 2298 ± 5%).

In addition to the intensities of the atomic columns of different types, we measured also distances between the atomic layers in the [001] direction (Figure 3b), i.e., perpendicular to the RP-type defects, and observed significant differences between the RP-type defects in nonperiodic sequences and Li_2_La_2_Ti_3_O_10_ phase. The La–E layers of the RP-type defects are contracted for about 11% (∼7.9 Å) in comparison to the equivalent spacing in the Li_2_La_2_Ti_3_O_10_ phase (8.961 Å, Figure 2c). Furthermore, the spacings between the La–E layers and the first La–C layer of the perovskite block are always slightly larger (∼4.0–4.1 Å) than the spacings in inside the perovskite blocks with *n* > 3 (∼3.9 Å). On the other hand, the Ti–E layers inside the RP-type defects are shifted slightly toward the La–E layers.

The observed relaxations of the La–E layers across the RP-defects, their higher standard deviation of intensities, and the presence of brighter contrast inside the Li-rich layers indicate a certain degree of ion exchange within the RP-defect layer (La–E – Ti–E – Li – Ti–E – La–E). To estimate the degree of ion exchange, we simulated HAADF-STEM images of models with different occupancies of the atomic layers inside the RP-layer. The structural models of the defects were based on Li_2_La_2_Ti_3_O_10_ (Figure 3c, left) with modified atomic positions (Figure 3c, right) as determined from the experimental HAADF-STEM image (Figure 3b). The “Only Shift” model included only the observed shift of lattice planes and no ion exchange. Image simulations of this model and other models with fully occupied Ti–E columns consistently yielded an intensity of the Ti–E columns that was too high compared to the intensity of the Ti–C columns (*I*_Ti–E_:*I*_Ti–C_). Therefore, models with lower occupancy of the Ti–E columns were prepared. Based on the variable intensity of the La–E columns, also various fractions of La atoms from these columns were repositioned to the Li-rich layer. Models with up to 20% Li atoms in the Li-rich layer (where twice the number of interstitial sites is available) randomly replaced with La and Ti from the two neighboring La–E and Ti–E layers were prepared. The La and Ti in these layers were simply replaced by Li. We are aware that Li atoms might occupy different interstitial sites^41^ or diffuse to the surrounding LLTO, however, the contribution of Li atoms to the contrast in HAADF-STEM images is negligible. Average compositions of the La–E, Ti–E, and Li-rich layers in the structural models used for image simulations are given in Table 1.

**Table 1:** Theoretical Compositions of the La–E, Ti–E, and Li-Rich Layers Comprising the RP-Type Defects in Models after Different Degrees of Ion Exchange

	only shift			10La-10Ti			20La-10Ti			15La-15Ti			10La-20Ti			20La-20Ti		
layer	La^3+^	Ti^4+^	Li^+^	La^3+^	Ti^4+^	Li^+^	La^3+^	Ti^4+^	Li^+^	La^3+^	Ti^4+^	Li^+^	La^3+^	Ti^4+^	Li^+^	La^3+^	Ti^4+^	Li^+^
La–E	1	0	0	0.9	0	0.1	0.8	0	0.2	0.85	0	0.15	0.9	0	0.1	0.8	0	0.2
Ti–E	0	1	0	0	0.9	0.1	0	0.9	0.1	0	0.85	0.15	0	0.8	0.2	0	0.8	0.2
Li-rich	0	0	2	0.2	0.2	1.6	0.4	0.2	1.4	0.3	0.3	1.4	0.2	0.4	1.4	0.4	0.4	1.2
Charge RP-layer	2–			1–			0.6–			0.5–			0.4–			0		
Composition RP-layer	[Li_2_O_2_]^2–^			[La_0.2_Ti_0.2_Li_1.6_O_2_]^1–^			[La_0.4_Ti_0.2_Li_1.4_O_2_]^0.6–^			[La_0.3_Ti_0.3_Li_1.4_O_2_]^0.5–^			[La_0.2_Ti_0.4_Li_1.4_O_2_]^0.4–^			[La_0.4_Ti_0.4_Li_1.2_O_2_]^0^		

The average charge of the Li-rich layer after exchange of a certain fraction of Li atoms with Ti and La can be calculated as (*x*·(3+) + *y*·(4+) + (2 – *x* – *y*) · (1+) + 2·(2−), where *x* and *y* are the fractions of La^3+^ and Ti^4+^ in the Li-rich layer, respectively. While the Li_2_–O_2_ layers in Li_2_La_2_Ti_3_O_10_ are negatively charged (2−), they become less negatively charged with an increasing exchange of Li^+^ with La^3+^ and Ti^4+^ and, theoretically, after an exchange with 20 at. % of La and 20 at. % of Ti (the “20La-20Ti” model), the layer would become electroneutral. At the same time, the average charge of the La–E layer decreases from 1+ to 0, whereas the reduced number of Ti atoms in the octahedral layer is most likely accompanied by the formation of oxygen vacancies and the formation of distorted octahedra. This is supported by the results of EELS analyses, which were used to confirm the presence of Li and Ti in LLTO and inside the RP-defects (Figure S7). The edge structure of Ti is similar in both areas, and the Ti-L_2_ and Ti-L_3_ peak splitting confirms that Ti is in a tetravalent state in LLTO and inside the RP-defect. The small difference in the edge structure was observed also by Zhu et al. (2020),^33^ who studied the structure of single atomic layer defects inside LLTO with similar structural characteristics as the RP-type defects in this study and they ascribed the small difference to the distortion of Ti-octahedra inside the defect layer.

Intensities of the atomic columns in image simulations of the models with different exchange rates and for different thicknesses were analyzed similarly to the experimental images. The results are graphically presented in Figure S8 and the intensity ratios and their comparison to the experimental values (image in Figure 3b) in terms of (average) absolute differences for 30, 20, and 15 nm thicknesses of the crystalline slab are given in Table S3. The best match is obtained for the 15 nm thick “10La-10Ti” model, and also simulations of the 20 and 15 nm thick “10La-20Ti” model fit well (Figure 3d). However, the overall thickness of the area where Figure 3b was acquired was determined by EELS to be around 30 nm (Figure S6) indicating that the sample contains some amorphous material on the surface. To evaluate the effect of the amorphous layer on the intensity ratios, we simulated the three models with the best fit with the addition of amorphous layers (above and below the crystalline part; see Figure S9 for details) to obtain the overall thickness of 30 nm. Simulation of the 15 nm thick “10La-20Ti” model with the addition of an amorphous layer shows improved fit to the experimental intensity ratios (Table 2).

**Table 2:** Comparison of Image Intensity Ratios in Image Simulations Showing the Closest Match with the Experimental Values for the HAADF-STEM Image Shown in Figure 3b

		*I*_La–C_/*I*_La–E_	*I*_Li_/*I*_La–E_	*I*_Ti–C_/*I*_La–E_	*I*_Ti–E_/*I*_La–E_	average absolute difference
		absolute differences to the reference				
Model “10La-10Ti”	15 nm (crystalline)	75.3	22.3	40.4	48.3	
		3.0	2.9	0.4	0.1	1.6
	30 nm (15 nm crystalline + 15 nm amorphous)	73.8	27.2	46.1	52.3	
		1.5	2.0	5.3	4.1	3.2
Model “10La-20Ti”	20 nm (crystalline)	71.5	28.3	42.5	52.4	
		0.8	3.2	1.7	4.2	2.5
	30 nm (20 nm crystalline + 10 nm amorphous)	73.5	29.9	44.7	53.7	
		1.2	4.8	3.9	5.5	3.8
	15 nm crystalline	72.4	23.9	35.4	46.6	
		0.1	1.3	5.4	1.6	2.1
	30 nm (15 nm crystalline + 15 nm amorphous)	71.8	28.9	41.1	46.8	
		0.5	3.7	0.3	1.4	1.5
Exp. image (Figure 3b)		72.3	25.2	40.8	48.2	reference

Comparison of image simulations of models with different compositions with experimental images (Figure 3b) suggests that up to 10% of La and up to 20% of Ti from the La–E and Ti–E layers exchange with Li in the RP-layer. The actual composition of different RP-defects may vary slightly, however, all models with a higher La exchange rate show worse fit indicating that the exchange of Ti is faster than that of La. With progressive ion exchange and out-diffusion of Li, the RP-layers become structurally unstable and progressively recrystallize to LLTO perovskite as described in the next chapter.

### Recrystallization of RP-Defects to LLTO Perovskite

**Figure 4:**
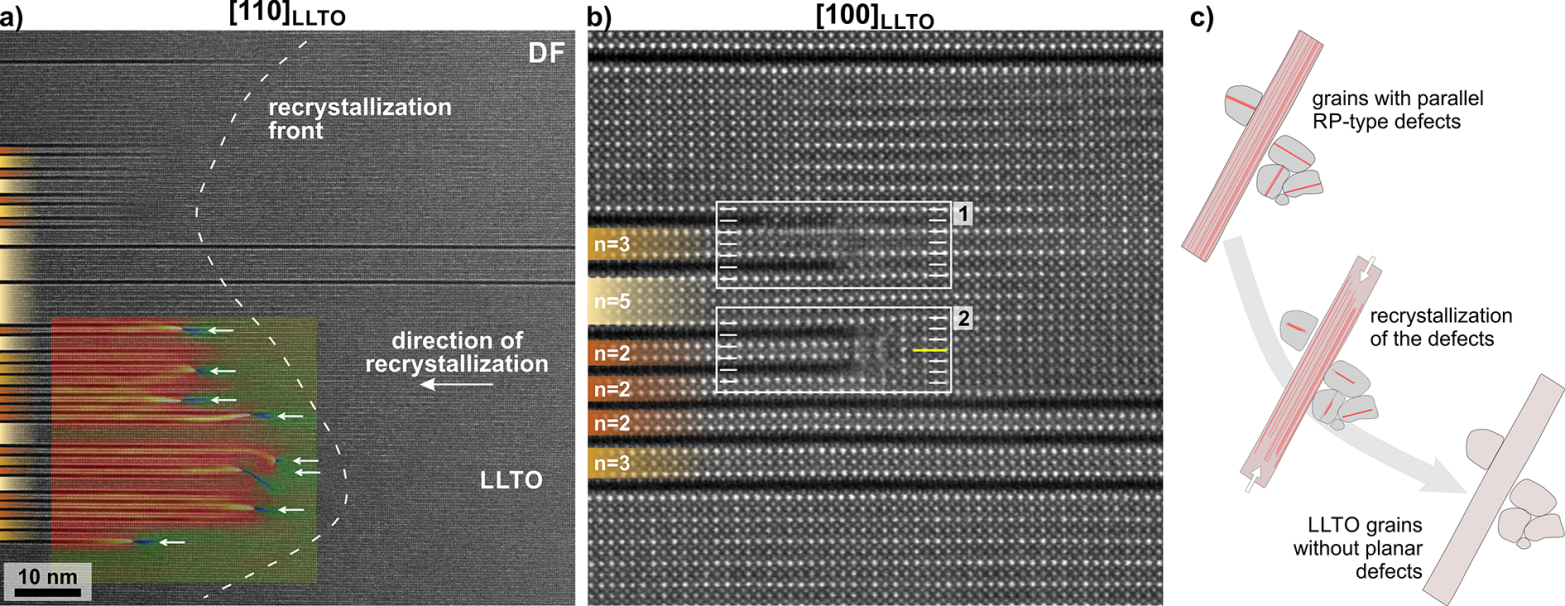
(a) Recrystallization of a nonperiodic lamella to LLTO perovskite. Arrows indicate the additional lattice planes in the newly formed LLTO. (b) Recrystallization of parallel RP-type defects to LLTO (1) without and (2) with the formation of an additional lattice plane (marked by the yellow line). Note that two parallel RP-type defects are needed for the recrystallization to cancel out the RP-type shift. (c) In the process of recrystallization, grains with RP-type lamellae are transformed into LLTO perovskite grains without planar defects.

The RP-type lamellae start to form in the early stage of microstructure development, even before significant densification of the sample. At around 1250 °C, when the sample is densified to the level suitable for preparation of TEM samples, the RP-defects in the nonperiodic sequences undergo significant internal ion exchange as described in the previous chapter. At the same time, some lamellae already start to recrystallize to LLTO perovskite, as shown in Figure 4a. The process may be described as topochemical conversion of layered 2D perovskite to 3D perovskite^42^ along [100] direction of Li_2_La_2_Ti_3_O_10_. Due to the mismatch between the nonperiodic sequences with RP-type defects and the newly forming LLTO, the transformation is accompanied by the formation of an additional lattice plane every few nanometers as identified by geometric phase analysis (GPA)^43^ in Figure 4a. High-resolution image of the recrystallization region along the [100] zone axis of LLTO shows that some RP-type lamellae recrystallize directly to LLTO and some with the formation of an additional lattice plane (Figure 4b). In the recrystallization process, the La atoms from (almost) fully occupied La-columns of the RP-defects (the La–E layers) rearrange to the A-sites of the newly formed LLTO perovskite (see the intensities of the A-sites in Figure S10) along with Li and vacancies. Excess Li can easily diffuse into the surrounding LLTO matrix due to its high mobility. In the areas where additional lattice planes are formed, the amount of Ti is slightly deficient, which is likely compensated by oxygen vacancies. During the recrystallization, the RP shift of two subsequent RP-defects is canceled out, and therefore the recrystallization of an even number of parallel defects is energetically not demanding. In the case of an odd number of RP-type defects, removal of the one remaining RP-defect would require recrystallization of a large LLTO domain, which would require more energy, and therefore, single planar defects occasionally remain inside the LLTO matrix and form isolated 2D defects. Such defects were described as single-atom-layer-traps (SALTs) by Zhu et al. (2020).^33^ They have shown that these defects limit Li ionic transport and degrade the total conductivity; therefore, recrystallization of as many defects as possible is preferred. Recrystallization of RP-type lamellae starts at the grain boundaries and progresses toward the interior of the grains. In this process, the grains are converted from LLTO grains with RP-type defects to normal LLTO perovskite grains as shown in Figure 4c. Assuming a comparable recrystallization rate, it is likely that the recrystallization of smaller LLTO grains with shorter and thinner in-grain lamellae is completed sooner than the recrystallization of the large platelike grains with thicker and longer RP-lamellae.

### Energy Aspects of Thermodynamic Phase Stability and Recrystallization

To understand the early formation of the Li_2_La_2_Ti_3_O_10_ phase and its further recrystallization to the LLTO perovskite, we performed first-principles calculations based on density functional theory to evaluate the thermodynamic phase stability. To simulate the experimental conditions, we followed the experimental precursor stoichiometry (Li:La:Ti = 11:15:25) for phase stability evaluation. Since the amount of O in the system could not be simply determined from the experimental precursors in an open system, the stoichiometry of O in the system was decided by finding the most stable combination of stable compounds with Li:La:Ti = 11:15:25 at the ground state, i.e., at 0 K. With the calculated formation energies of all of the known stable compounds in the Li–La–Ti–O quaternary system, the most stable combination of compounds was determined to be 2.5 Li_2_TiO_3_ + 1.5 Li_4_Ti_5_O_12_ + 7.5 La_2_Ti_2_O_7_. Therefore, the system was presumed to be with the Li:La:Ti:O = 11:15:25:78 stoichiometry for thermodynamic phase stability evaluations.

First, we assume that the main reaction product is Li_0.167_La_0.61_TiO_3_ perovskite with the La:Ti ratio as in the starting powder. A Li_3_La_11_Ti_18_O_54_ model was built to simulate the LLTO phase. The reaction of the most stable combination of compounds reacting into Li_0.167_La_0.61_TiO_3_ LLTO perovskite could be shown as following eq 1:
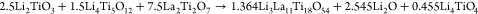
1

Further, we predict Li_0.33_La_0.56_TiO_3_ with a lower La:Ti ratio as the main reaction product. A Li_6_La_10_Ti_18_O_54_ model was built to simulate this type of LLTO perovskite. In this case, the reaction of the most stable combination of compounds reacting into Li_0.33_La_0.56_TiO_3_ could be shown as eq 2:
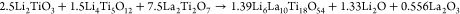
2

As presented in the previous experimental sections, a Ti-deficient LLTO perovskite would form via the precursor RP-type phase, given that the stoichiometry of La:Ti for the Li_0.33_La_0.56_TiO_3_ perovskite and the RP-type phase are 5:9 and 3:5, respectively. Tanaka et al. (2003) reported that charge-compensating vacancies could form in perovskite material more easily compared to only cation vacancies in a reducing environment.^44^ Therefore, with the Ti deficiency and the excess Li in the precursor RP-type phase, the vacancies created in the Ti-deficient LLTO model should follow a Ti:O = 1:2 ratio. To mimic the Ti-deficient LLTO perovskite, a LLTO perovskite supercell model with La:Ti = 10:17 stoichiometry was created, in which a Ti vacancy and two corresponding O vacancies to maintain the charge neutrality were created based on the pristine Li_6_La_10_Ti_18_O_54_ model. The formation energy of the Ti- and O-deficient Li_0.33_La_0.56_TiO_3_ perovskite was calculated with the Li_6_La_10_Ti_17_O_52_ model, which was one of the main reaction products. For such a case, according to mass conservation, the most stable reaction products would be 1.47 Li_6_La_10_Ti_17_O_52_ + 1.088Li_2_O + 0.147La_2_O_3_, as shown in eq 3:
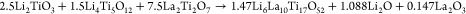
3

On the other hand, the other main reaction product is the Li_2_La_2_Ti_3_O_10_ RP-type phase. For such a case, the most stable reaction products would be 5.5 Li_2_La_2_Ti_3_O_10_ + 4.5TiO_2_ + 2 La_2_Ti_2_O_7_, as shown in eq 4:
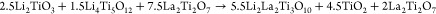
4

Combining eqs 1, 2, and 3 with eq 4, the Li_2_La_2_Ti_3_O_10_ to different LLTO perovskite phases, including Li_0.167_La_0.61_TiO_3_ (Li_3_La_11_Ti_18_O_54_), Li_0.33_La_0.56_TiO_3_ (Li_6_La_10_Ti_18_O_54_), and Ti- and O-deficient Li_0.33_La_0.56_TiO_3_ (Li_6_La_10_Ti_17_O_52_), reaction energies can be determined:
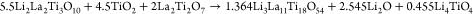
5Reaction energy = 3.86 eV.
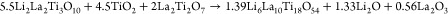
6Reaction energy = 3.52 eV.
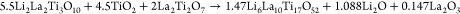
7Reaction energy = 6.53 eV.

The positive energies of eqs 5, 6, and 7 indicate that Li_2_La_2_Ti_3_O_10_ is the most stable phase at 0 K (low temperatures). The formation of the Li_0.33_La_0.56_TiO_3_ perovskite becomes more favorable during high-temperature heat treatment considering the Li and O loss according to the Le Chatelier principle, which matches well with the experimental results. Furthermore, the lower reaction energy of the Li_0.33_La_0.56_TiO_3_ perovskite (3.52 eV) as compared to the Li_0.167_La_0.61_TiO_3_ (3.86 eV) and Ti- and O-deficient Li_0.33_La_0.56_TiO_3_ (6.53 eV) indicates that direct crystallization of Li_0.33_La_0.56_TiO_3_ LLTO is preferential compared to LLTO perovskites with other La:Ti ratios. All phases should exhibit similar electronic conductivity, as the Ti and O vacancies in the Ti- and O-deficient LLTO resemble a Schottky defect in TiO_2_,^45^ where these vacancies do not introduce an extra electron. A band gap for Li_0.33_La_0.56_TiO_3_, Li_0.167_La_0.61_TiO_3_, and Ti- and O-deficient Li_0.33_La_0.56_TiO_3_, calculated from the density of states (DOS), were 1.67, 1.92, and 1.96 eV, respectively (Figure S11). Similar values were also obtained by Chouiekh et al. (2023).^46^

### The Influence of Transient RP-Type Defects on Microstructure Development and Properties of LLTO-Based Solid Electrolytes

**Figure 5:**
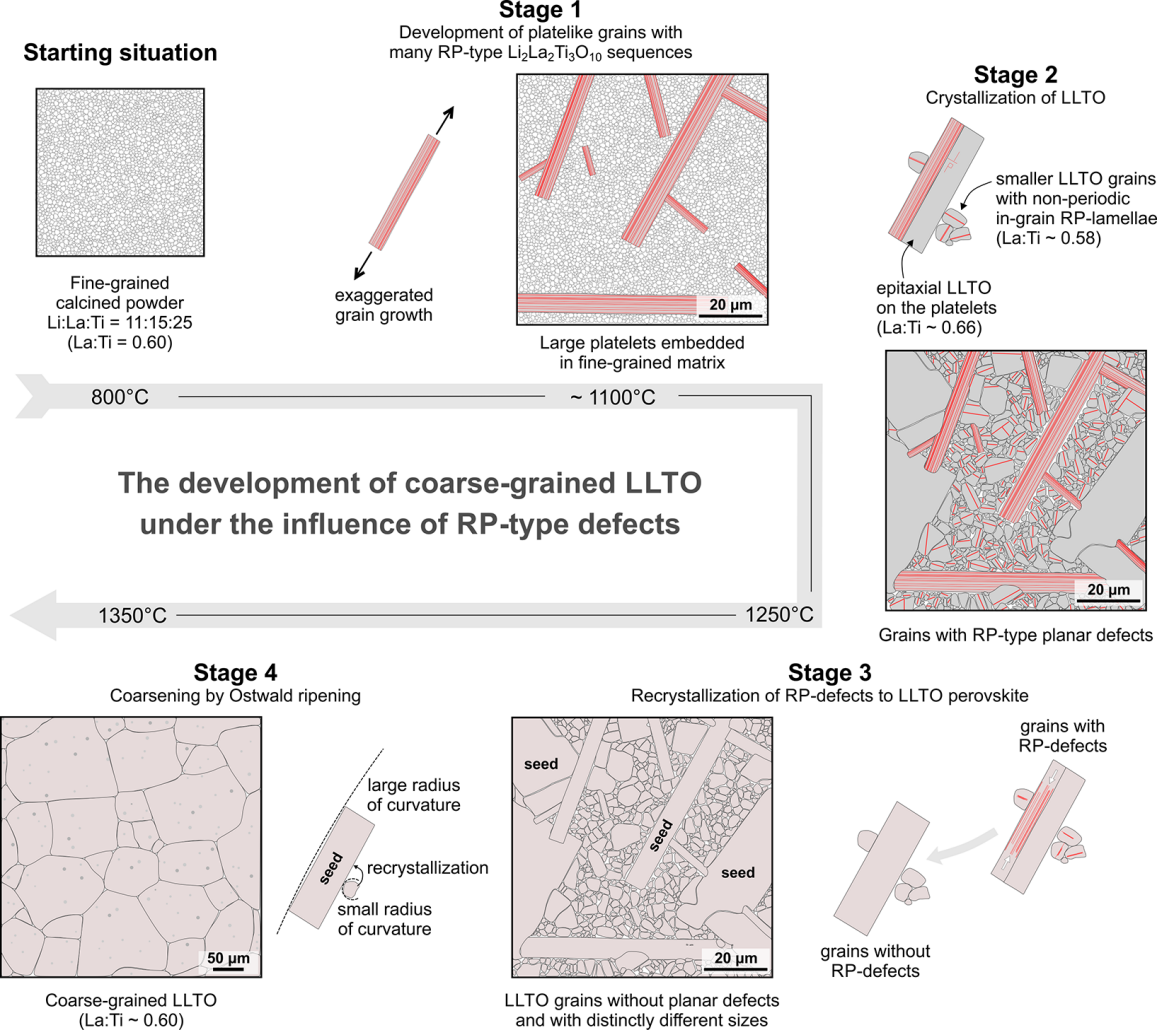
Schematic presentation of phases during microstructure development of coarse-grained LLTO ceramics under the influence of RP-type defects. Detailed description in text.

In fine-grained calcined powder with the starting Li:La:Ti ratio of 11:15:25 (*Starting situation* in Figure 5), the formation of Li_2_La_2_Ti_3_O_10_ phase is energetically more favorable compared to the LLTO perovskite (eqs 5–7), and therefore, microstructure development starts with the formation of grains that contain dense sequences of the layered RP-type Li_2_La_2_Ti_3_O_10_ phase (Figure 2). The formation of this phase is preferred in the beginning when the phase equilibrium is shifted toward the Li- and La-rich Li_2_La_2_Ti_3_O_10_ phase. Due to the close structural relationship between the Li_2_La_2_Ti_3_O_10_ and LLTO perovskite, sequences of the RP-type Li_2_La_2_Ti_3_O_10_ phase are frequently interrupted by perovskite blocks with variable thicknesses (Figure 3). Grains with many parallel RP-defects that form in the early stages of microstructure development exhibit fast and preferential growth in the direction of the RP-layers, i.e., the [100] direction of the Li_2_La_2_Ti_3_O_10_ phase, and develop into large thin anisotropic platelets with a thickness of up to 10 μm and lengths that sometimes exceed 100 μm (*Stage 1* in Figure 5). Such anisotropic grain growth is typical for unconstrained growth of phases with a layered structure like the Bi_4_Ti_3_O_12_ Aurivillius phase^47^ which has similar crystal structure as Li_2_La_2_Ti_3_O_10_.

With increasing sintering temperature, microstructure development continues with preferential crystallization of LLTO perovskite (*Stage 2* in Figure 5) with a La:Ti ratio around 0.58. Some LLTO grows epitaxially on the large platelets and forms large LLTO areas with single 2D defects along the equivalent 100 directions of the perovskite structure, whereas most of LLTO crystallizes in the form of smaller grains between the large platelets. The presence of thin nonperiodic RP-type lamellae is characteristic also of all smaller LLTO grains. These lamellae also show exaggerated growth along the layers and sometimes even penetrate the neighboring grains (Figure 1f).

Characteristic of exaggerated growth of grains with inherent planar defects is that these grains grow until they collide with other equivalent grains; then their growth is stopped. In our sample, this effect is observed in the large platelets, as well as in the small LLTO grains with thin in-grain lamellae. The thin RP-type lamellae inside the smaller LLTO grains clearly precede the growth of the matrix LLTO that barely follows the pace of the lamella. This process was described as polytype-induced grain growth in other perovskites and oxides, where the formation of planar defects with locally different atomic structure and chemical compositions is observed, e.g., BaO-doped CaTiO_3_,^48^ BaTiO_3_ sintered in reducing atmosphere,^49^ SrO-doped SrTiO_3_,^32^ and ZnO doped with SnO_2_, TiO_2_ or Sb_2_O_3_.^50^

At the end of *Stage 2* at around 1250 °C, the microstructure consists of large platelets with many RP-type defects, some with epitaxially overgrown LLTO perovskite, and these grains are surrounded by many small LLTO grains with thinner RP-type lamellae (bimodal grain size distribution, Figure 1a). If the RP-type defects inside the LLTO grains were thermally stable, further recrystallization would be prevented. However, in this system, the RP-type defects become unstable at around 1250 °C and start to recrystallize to the LLTO perovskite. The process starts with ion exchange inside the RP-layers (Li-rich layers), where the highly mobile Li atoms diffuse out of the Li-rich RP-defects and the La and Ti atoms from the neighboring atomic layers replace the Li atoms inside the Li-rich layer. The initially highly charged (001) atomic layers of the Li_2_La_2_Ti_3_O_10_ phase are gradually becoming electroneutral (Figure 3) and, at the same time, structurally unstable. In the next stage of microstructure development, the RP-sequences steadily recrystallize to the LLTO perovskite (*Stage 3* in Figure 5). Oriented topotactic recrystallization of the RP-type sequences may locally lead to the formation of Ti- and O-deficient LLTO perovskites (eq 7). After recrystallization of the RP-type defects to LLTO, the grains lose their reinforcement and become normal LLTO grains without planar defects. The result of this process are grains with two significantly different grain sizes and morphologies (bimodal grain size distribution). The large faceted platelets have incomparably larger radii of curvature and act as seed grains for recrystallization of the smaller more isometric LLTO grains with smaller curvature via the Ostwald ripening mechanism (*Stage 4* in Figure 5). Since recrystallization of the larger seed grains may take longer than the recrystallization of the smaller grains, the processes of recrystallization of the RP-type defects and recrystallization of the smaller grains onto the seed grains may overlap. Recrystallization of the RP-defects within the smaller grains is a prerequisite for the onset of the Ostwald ripening mechanism. This process leads to the development of a final microstructure composed of large LLTO grains, some measuring up to 250 μm in diameter. According to SEM/EDXS analyses, the grains have a homogeneous La:Ti ratio of around 0.6, which is close to the ratio in the starting powder (Figure 1g). This implies the recrystallization of regions with different La:Ti ratios, also the Ti-deficient regions that form during the recrystallization of the RP-lamellae, to homogeneous LLTO (at the level of SEM/EDXS) during prolonged sintering at 1350 °C. As shown in our previous study,^30^ the coarse-grained LLTO has about 1 order of magnitude higher total ionic conductivity (10^–4^ S/cm) in comparison to the LLTO with *x* = 0.11 (10^–5^ S/cm) due to the lower fraction of grain boundaries and these values may be further improved by doping^51−55^ and using optimized processing conditions, e.g., sintering in a moisture-free or oxygen-rich atmosphere.^27,56^

## Conclusions

In this work, we studied microstructure development in LLTO solid electrolyte with a starting La:Ti ratio of 0.60 and a high excess addition of Li. HAADF-STEM analyses in combination with theoretical calculations confirm the preferential formation of the Li_2_La_2_Ti_3_O_10_ phase at lower temperatures in comparison with the LLTO perovskite. This results in the formation of grains with periodic and nonperiodic RP-type Li_2_La_2_Ti_3_O_10_ phase sequences, which exhibit exaggerated growth along the RP-type layers and develop into large anisotropic platelike grains with thicknesses of up to 10 μm and more than 100 μm in length. At higher sintering temperatures, microstructure development continues with the crystallization of LLTO perovskites with single RP-type defects and thinner in-grain RP-lamellae. At around 1250 °C, the RP-type sequences become thermally unstable and gradually recrystallize to the LLTO perovskite. Recrystallization occurs via ion exchange between the Li-rich RP-layers and the neighboring Ti and La layers, where up to 10% of La^3+^ and 20% of Ti^4+^ replace Li^+^ in the Li-rich RP-layers. Consequently, the RP-layers become more electroneutral but also structurally unstable and recrystallize to the LLTO perovskite. After recrystallization of RP-type defects in both larger platelike grains and smaller LLTO grains, the large grains act as seeds for further recrystallization by Ostwald ripening. The self-seeded microstructure development is an alternative approach to the formation of coarse-grained LLTO solid electrolytes with a low fraction of resistive grain boundaries.

## Materials and Methods

### Synthesis Procedure

Lithium lanthanum titanate was prepared by mixing Li_2_CO_3_ (99.998%, Alfa Aesar, Massachusetts, USA), La_2_O_3_ (99.99%, Alfa Aesar, Massachusetts, USA), and TiO_2_ (≥99.9%, Sigma-Aldrich, St. Louis, USA) powders with a Li:La:Ti atomic ratio of 11:15:25. The composition corresponds to Li_3*x*_La_2/3–*x*_□_1/3–2*x*_TiO_3_ phase with *x* = 0.06 (Li_0.2_La_0.6_TiO_3_) and 142 at. % excess of Li. Relative to Li_0.33_La_0.56_TiO_3_ (*x* = 0.11), which is the LLTO perovskite with the highest reported Li ion conductivity, the starting powder contains around 7 at. % excess of La and 25 at. % excess of Li.

The starting powders were weighed and homogenized in a planetary ball mill at 200 rpm for 0.5 h with ZrO_2_ balls as the medium by using ethanol. La_2_O_3_ powder was treated at 800 °C for 10 h before weighting to remove the absorbed water. After the homogenized mixture was dried at 120 °C, the obtained powder was pressed in pellets with a diameter of 10 mm at a pressure of 100 MPa and calcined at 800 °C for 8 h in a tube furnace. The calcined pellets were crushed, milled, dried, pressed in pellets, and sintered at 1250 °C/12 h and 1350 °C/12 h in air.

### Electron Microscopy Characterization Techniques

Microstructure analyses were performed on thermally etched (1150 °C in air for 15 min) cross sections using a scanning electron microscope (SEM; Thermo Fisher Quanta 650 ESEM, Massachusetts, USA) with a thermionic electron source.

Samples for scanning transmission electron microscopy (STEM) were prepared by cutting a 3 mm disk from the ceramic pellet, mechanical thinning to ∼100 μm, dimpling to ∼20 μm in the disc center (Dimple grinder, Gatan Inc., Warrendale), and finally, ion milling to perforation using 3.8 keV Ar ions at an angle of 8° from both sides (PIPS 691, Gatan Inc., Pleasanton, USA). After perforation, the energy was gradually lowered, finally to 500 eV for 5 min to minimize the thickness of the amorphous surface layer. STEM analyses were performed using a probe-corrected atomic-resolution microscope (JEOL ARM200 CF, Jeol Ltd., Tokyo, Japan) operated at 200 kV and equipped with a high-angle annular dark-field (HAADF) detector with inner and outer semiangles of 68 and 180 mrad, respectively. EELS spectra were acquired using a Gatan DualEELS Quantum ER spectrometer. Samples for STEM analyses were coated with 2 nm of amorphous carbon (PECS 682, Gatan Inc., Pleasanton, USA) to prevent charging under the electron beam.

### Quantitative Analysis of HAADF-STEM Images

For quantitative analysis of experimental atomic-scale HAADF-STEM images, the atomic column intensities were normalized by subtracting the detector background signal.^57^ The local maxima in the images were determined using the *Find Maxima* algorithm in ImageJ. Pixel intensities of each atomic column were integrated within an approximated 2D Gaussian profile.^58^ The mask size for the determination of atomic column intensity was set approximately at full width at half-maximum (fwhm) of the atomic column intensity profile.

Simulations of HAADF-STEM images were carried out using quantitative image simulation software QSTEM with multislice method and frozen phonon approximation.^59^ The following microscope parameters were used: acceleration voltage 200 kV, chromatic aberration *Cc* 1.1 mm, probe convergence semiangle 24 mrad, annular detector angular range from 68 to 180 mrad, and energy spread dE = 0.4 eV. Thirty phonon configurations at a temperature of 300 K were calculated with included thermal diffuse scattering (TDS), which causes inelastic phonon excitation.

Crystal models for image simulations were constructed based on the crystal structures of Li_2_La_2_Ti_3_O_10_^60^ and visualized using Vesta.^61^ A Starting model of the structure with thicknesses of 510.9 Å (35644 atoms) was prepared in XYZ format. The starting model was initially simulated in its original form. Further, the model was modified in terms of atomic column positions (e.g., relaxation across the RP defects) and composition (different ionic exchange rates in the atomic columns next to the RP defects) to replicate the specific features observed in the experimental images. The absolute intensities of the individual atomic columns in the simulated HAADF images were determined using the same procedure as for the experimental images.

### Laser Ablation Inductively Coupled Plasma Mass Spectrometry (LA-ICP-MS)

LA-ICP-MS is a technique employed for the high-precision elemental analysis of solid samples. After the sample’s surface is ablated with a pulsed focused laser beam, the particles are ionized in an inductively coupled plasma and subsequently transferred to the mass spectrometer detector, where ions are separated according to their mass-to-charge ratio. The instrumental setup used in this work for LA-ICP-MS measurements was comprised of a laser ablation system (193 nm ArF* excimer; Analyte G2 Teledyne Photon Machines Inc., Bozeman, MT). The LA-system was equipped with a standard active two-volume ablation cell (HelEx II), including the Aerosol Rapid Introduction System (ARIS, Teledyne CETAC Technologies) for fast aerosol washout. The LA unit was coupled to a quadrupole ICP-MS instrument (Agilent 7900x, Agilent Technologies, Santa Clara, CA). Ablation parameters were as follows: laser energy density, 3.0 J cm^–2^; repetition rate, 250 Hz; beam diameter, 3 μm; dosage 10 and total acquisition time for ICP-MS acquisition was 0.04 s (with corresponding dwell times for specific nuclides: ^7^Li, 15 ms; ^47^Ti, 8 ms; and ^139^La, 8 ms). Other parameters were based on model predictions for the fastest possible mapping times, avoidance of aliasing, minimal blur, and maximal S/N ratios.^62,63^ The ablated material was transported from the ablation cell to the ICP using helium as a carrier gas, and argon was added as a makeup gas before the torch of the ICP. Data processing and image analysis were performed using the software packages HDIP (Teledyne Photon Machines Inc., Bozeman, MT) and ImageJ.

### First-Principle Calculations

First-principles computations based on density functional theory (DFT)^64^ were used for thermodynamical phase stability evaluation and density of states computation. Perdue–Burke–Ernzerhof (PBE) Generalized Gradient Approximation (GGA) exchange correlation^65^ and Projector Augmented-Wave (PAW) pseudopotential^66^ implanted in Vienna Ab initio Simulation Package (VASP)^67,68^ were adopted for the first-principles computations. The LLTO models (Li_3_La_11_Ti_18_O_54_ and Li_6_La_10_Ti_18_O_54_) were built according to experimental XRD results,^69,70^ and the Ti- and O-deficient LLTO atomic model was built by removing 1 Ti atom and 2 O atoms from Li_0.33_La_0.56_TiO_3_. Atomic models of Li_2_La_2_Ti_3_O_10_ and stable compounds in the Li–La–Ti–O quaternary system were taken from the Materials Project database.^71^ All of the computations were converged to 10^–4^ eV energy convergence and 10^–2^ eV/Å force convergence. The *k-point* meshes used for Li_0.167_La_0.61_TiO_3_, Li_0.33_La_0.56_TiO_3_, and Ti- and O-deficient Li_0.33_La_0.56_TiO_3_ total energy and density of states computations were 2 × 2 × 3 and 4 × 4 × 6, respectively.

Phase stability evaluation was conducted by finding the most stable combination of compounds within the Li:La:Ti:O = 11:15:25:78 stoichiometry by calculating the decomposition energies of the main reaction products, including Li_0.167_La_0.61_TiO_3_ Li_0.33_La_0.56_TiO_3_, Ti- and O-deficient LLTO, and Li_2_La_2_Ti_3_O_10_, by eq 8:^72^
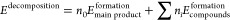
8where *E*^decomposition^ is the decomposition energy; *E*_main product_^formation^ and *E*_compounds_^formation^ are the formation energies of main reaction products (Li_0.167_La_0.61_TiO_3_, Li_0.33_La_0.56_TiO_3_, Ti- and O-deficient Li_0.33_La_0.56_TiO_3_, and Li_2_La_2_Ti_3_O_10_) and stable compounds in the Li–La–Ti–O quaternary system, respectively. *n*_0_ and *n*_*i*_ are the coefficients of the compounds that can fulfill the stoichiometry (Li:La:Ti:O = 11:15:25:78).

## Supporting Information Available

Powder XRD pattern, LA-ICP-MS maps, SEM/EDXS analysis of calcined powder, EELS spectra and map, STEM images, simulated images with intensity profiles, models for simulations, absolute atomic column intensities with intensity ratios, and average absolute differences for experimental and simulated images (PDF)

## Abbreviations

BF-STEM: bright-field scanning transmission electron microscopy
BSE (image): backscattered image
DFT: density functional theory
EELS: electron energy loss spectroscopy
fwhm: full width at half maximum
GB: grain boundary
HAADF-STEM: high-angle annular dark-field scanning transmission electron microscopy
ICP-OES: inductively coupled plasma optical emission spectrometry
LA-ICP-MS: laser ablation inductive coupled plasma mass spectroscopy
LLTO: lithium lanthanum titanate
RP: Ruddlesden–Popper
TDS: thermal diffuse scattering
