# Supplementary material for: Transient Ruddlesden–Popper-Type Defects and Their Influence on Grain Growth and Properties of Lithium Lanthanum Titanate Solid Electrolyte
Source: ACS Nano. 2024 Apr 9;18(16):10850–62. doi: 10.1021/acsnano.4c00706 (PMC11044694; doi:10.1021/acsnano.4c00706)
Supplement: Supplementary file 1 — nn4c00706_si_001.pdf [file nn4c00706_si_001.pdf]

Supporting Information for

Transient Ruddlesden-Popper-type Defects and Their Influence on Grain Growth and Properties of Lithium Lanthanum Titanate Solid Electrolyte

Petruša Borštnar[1,2], *Goran Dražić*[3], *Martin Šala*[4], *Che-an Lin*[5], *Shih-kang Lin*[5,6,7,8], *Matjaž Spreitzer*[1], *Nina Daneu*[1]*

[1]Advanced Materials Department, Jožef Stefan Institute, Jamova cesta 39, Ljubljana, Slovenia

[2]Jožef Stefan International Postgraduate School, Jamova cesta 39, Ljubljana, Slovenia

[3]Department of Materials Chemistry, National Institute of Chemistry, Hajdrihova 19, Ljubljana, Slovenia

[4]Department of Analytical Chemistry, National Institute of Chemistry, Hajdrihova 19, Ljubljana, Slovenia

[5]Department of Materials Science and Engineering, National Cheng Kung University, Tainan 70101, Taiwan

[6]Hierarchical Green-Energy Materials (Hi-GEM) Research Center, National Cheng Kung University, Tainan 70101, Taiwan

[7]Program on Smart and Sustainable Manufacturing, Academy of Innovative Semiconductor and Sustainable Manufacturing, National Cheng Kung University, Tainan 70101, Taiwan

[8]Core Facility Center, National Cheng Kung University, Tainan 70101, Taiwan

*nina.daneu@ijs.si

Table of contents

Supplementary figures

Figure S1	Powder XRD pattern of the ceramics after sintering at 1250 °C.
Figure S2	LA-ICP-MS maps of samples after sintering at 1250 °C and 1350 °C.
Figure S3	Low-magnification BF-STEM image of in-grain lamella and single RP-defects in the surrounding LLTO matrix grain.
Figure S4	SEM/EDXS analysis of calcined LLTO powder.
Figure S5	Image simulations and intensity profiles for the $Li_2La_2Ti_3O_{10}$ model in [100] and [110] zone axes.
Figure S6	HAADF-STEM image of a thicker RP-lamella and EELS map for the determination of the thickness of the region.
Figure S7	HAADF-STEM image of non-periodic RP-type defects in contact with LLTO and corresponding EELS analysis.
Figure S8	Atomic column intensity ratios for models with different exchange rates and thickness from 10 to 50 nm and image simulations for models with a thickness of 20.9 nm.
Figure S9	Structural model with "10La-20Ti" exchange rate and thickness of 15 nm and the same model with the addition of amorphous layers.
Figure S10	HAADF-STEM image of the recrystallization region with overlaid atomic column intensities.
Figure S11	Density of states for the three LLTO models.

Supplementary tables

Table S1	ICP-OES results of LLTO samples after different thermal treatments.
Table S2	Absolute atomic column intensities and intensity ratios of the periodic $Li_2La_2Ti_3O_{10}$ sequence and simulations with different thicknesses along [100] and [110] zone axes.
Table S3	Average atomic column intensities with relative deviations and intensity ratios for non-periodic sequence and models with different exchange rates and thickness of 30, 20, and 15 nm.

Supplementary figures

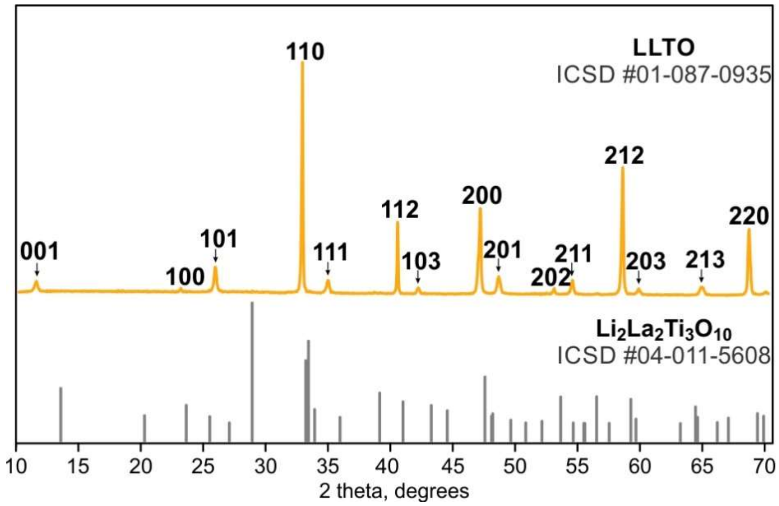

Figure S1. XRD pattern of LLTO ceramics with starting Li:La:Ti ratio of 11:15:25 after sintering at 1250 °C (above) showing tetragonal LLTO modification with strong superstructure peaks (marked with arrows). The sample does not contain any secondary phases. The fraction of the $Li_2La_2Ti_3O_{10}$ phase in the sample (characteristic peaks shown below) is below the XRD detection limit.

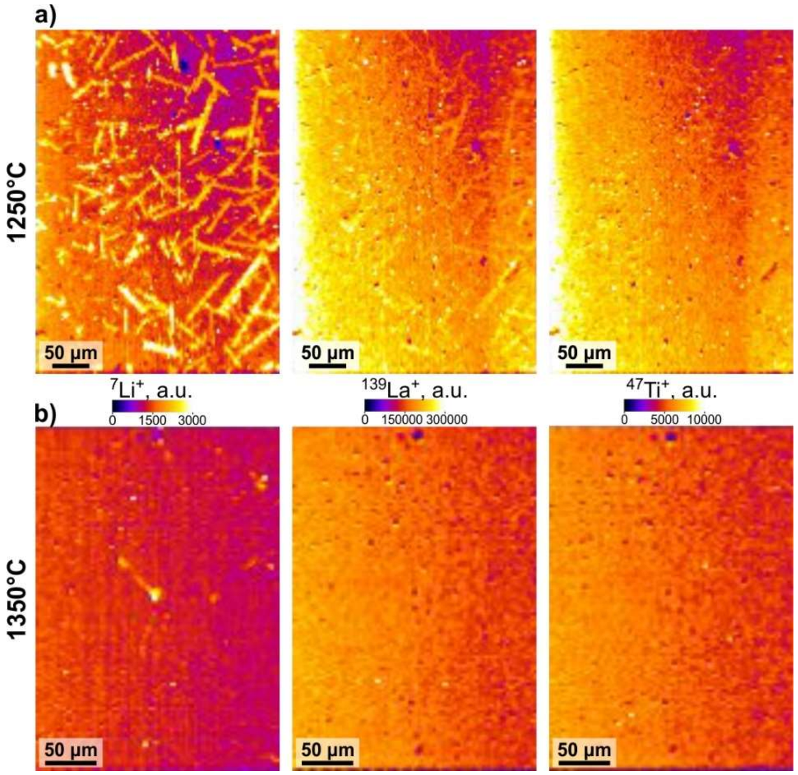

Figure S2. LA-ICP-MS analyses of the samples after sintering at (a) 1250 °C and (b) 1350 °C. Enrichment of the platelike grains with Li and La after sintering at 1250 °C can be observed. After sintering at 1350 °C, all elements are homogeneously distributed in the sample. The larger-scale color differences (e.g. in the upper right part of the 1250 °C maps and in the direction from left to right in the 1350 °C) stem from uneven sample height.

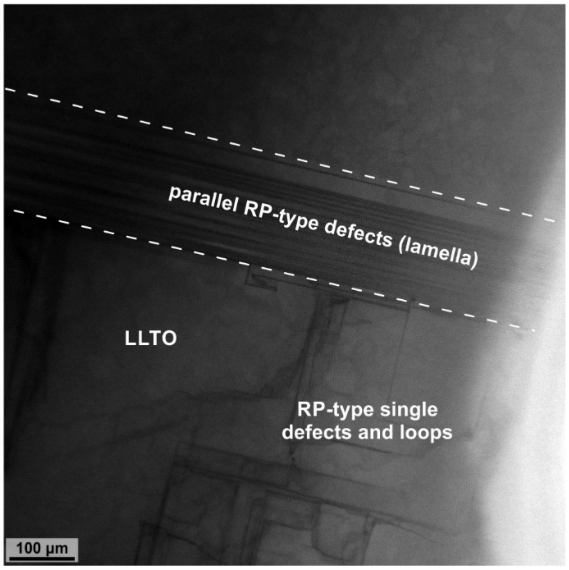

Figure S3. Single RP-defects along the equivalent {100} directions of the perovskite lattice are common in the matrix LLTO next to the in-grain lamellae composed of many parallel RP-type defects.

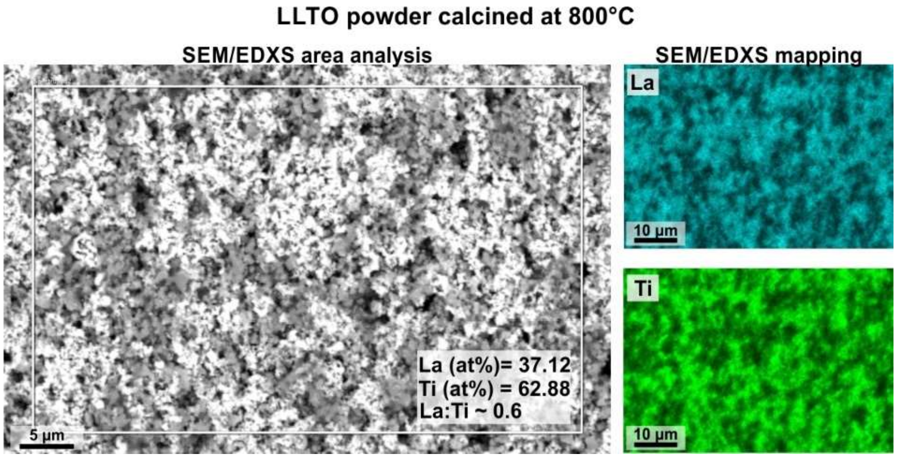

Figure S4. SEM/EDXS area analysis of LLTO starting powder calcined at 800 °C for 10 h. According to area analysis, the average La:Ti ratio is around 0.6. EDS mapping shows that La and Ti are uniformly distributed in the sample.

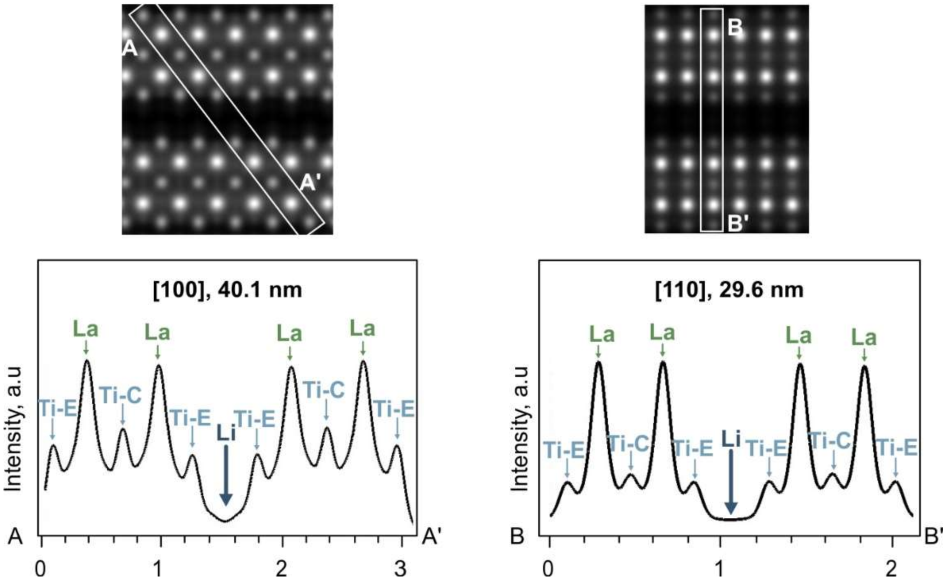

Figure S5. Intensity profiles across two pseudo-perovskite blocks in a simulation of $Li_2La_2Ti_3O_{10}$ in [100] and [110] zone axes for thickness 40 nm and 30 nm, respectively.

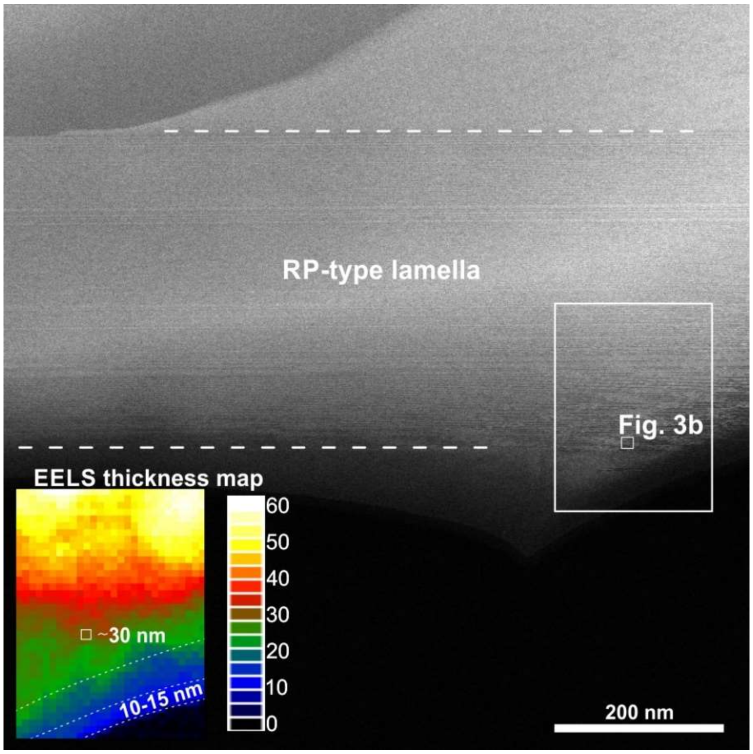

Figure S6. A thicker RP-lamella where the HR-HAADF-STEM image (stack) shown in Fig. 3b was acquired. EELS mapping was used for the determination of the thickness of the region. A thin part of amorphous regions with a thickness from 10 to 15 nm is present at the edge of the sample. The overall thickness of the area where the HAADF-STEM stack was acquired is about 30 nm (29.6 nm). The thickness includes the crystalline part as well as the amorphous layers on the top and below the crystalline part.

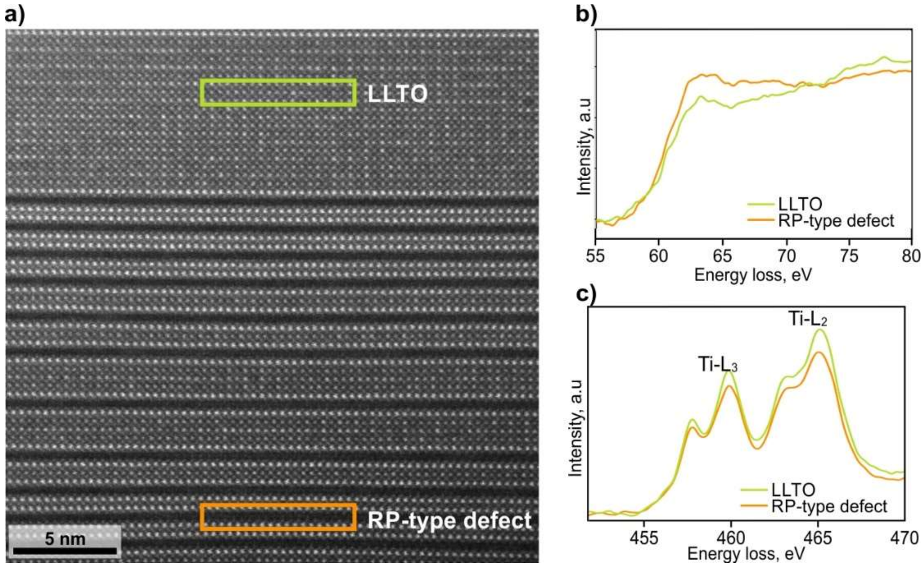

Figure S7. (a) HAADF-STEM image of non-periodic RP-type defects (below) in contact with LLTO (above). (b) EELS analyses of the RP-type defect and LLTO showing the Li *K* and Ti $L_{2,3}$ edge. The Ti-$L_{2,3}$ white lines in both spectra are positioned at the same energy indicating the presence of Ti in 4+ oxidation state in both areas.

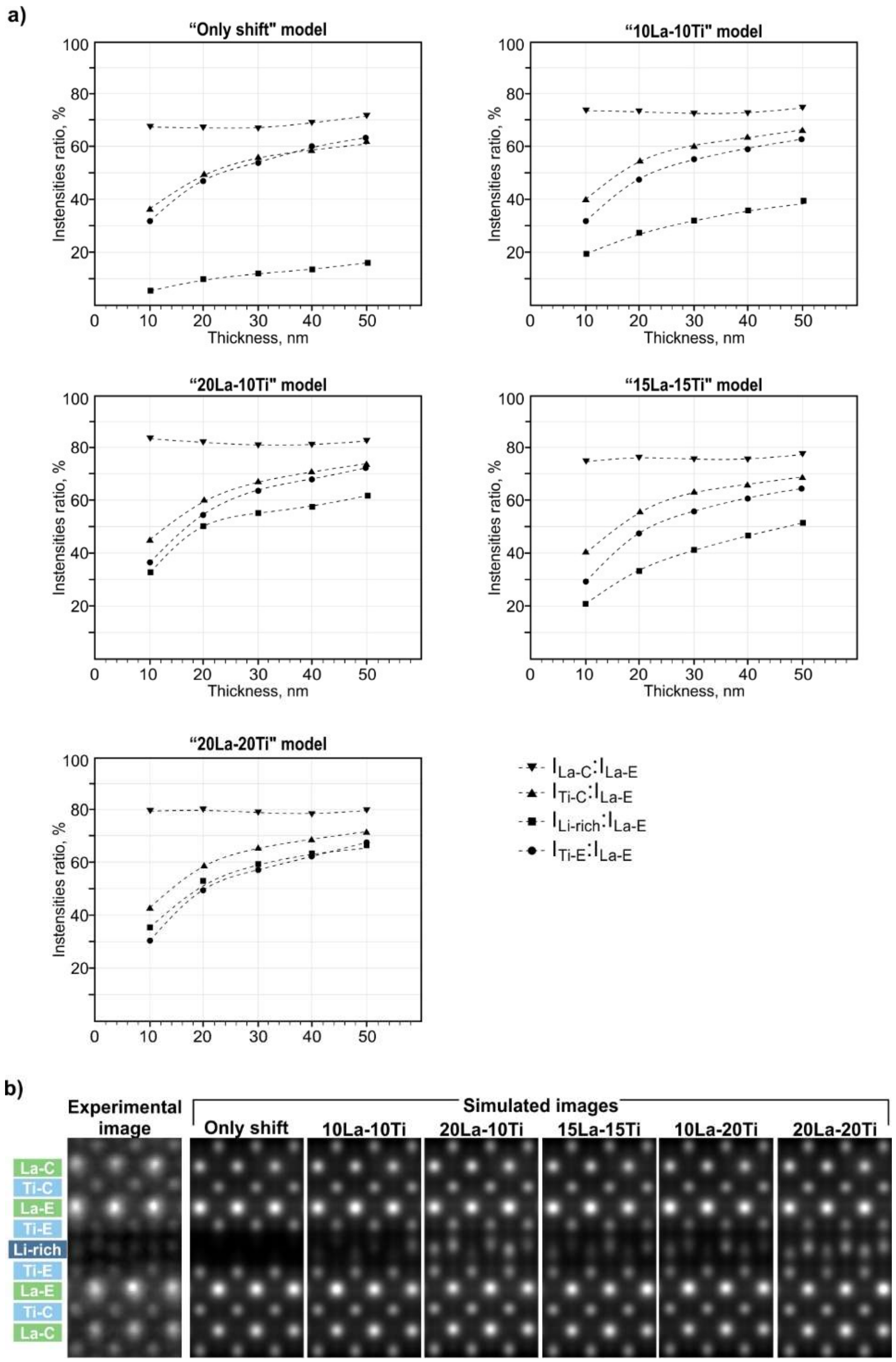

Figure S8. (a) Intensity ratios of the atomic columns in image simulations of the models with different exchange rates (given in Table 1) and for thicknesses from 10 to 50 nm. (b) Comparison of the experimental image with image simulations of different structural models for the thickness of 20.9 nm.

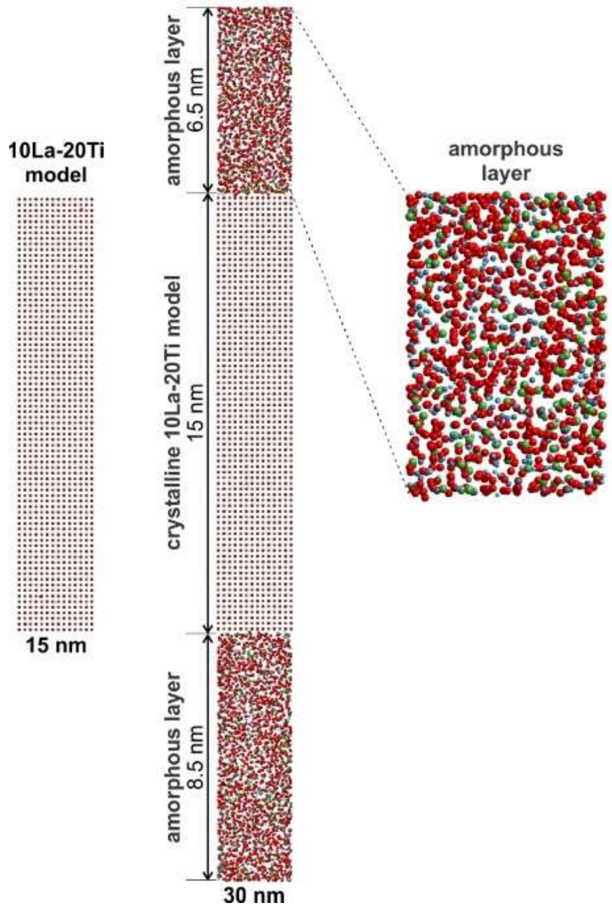

Figure S9. Structural model with '10La-20Ti' exchange rate and thickness of 15 nm and model with the addition of an amorphous layer with a total thickness of 15 nm. The amorphous layers were prepared by removing half of the atoms (randomly) in the crystalline $Li_2La_2Ti_3O_{10}$ model to reduce the density, followed by a random displacement of each individual atom. The bottom amorphous layer is thicker because it includes 2 nm of amorphous carbon sputtered on the surface of the sample. Note that the focus is always set on the crystalline part of the model. The comparison of intensity ratios between the two models is presented in Table 2.

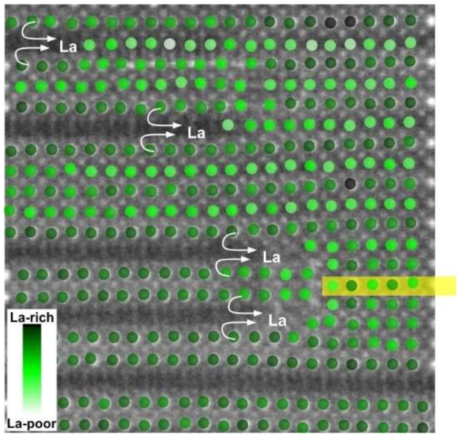

Figure S10. Enlarged section from Figure 4b with overlaid atomic column intensities showing the local rearrangement of La atoms from La-E layers of RP-defects to LLTO perovskite. Recrystallization can occur directly to LLTO or by the formation of an additional lattice plane. Note that two parallel RP-type defects are needed for the recrystallization to cancel out the RP-type shift.

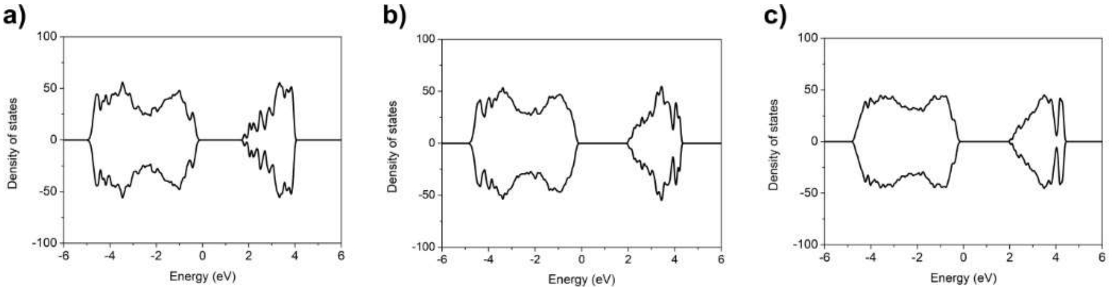

Figure S11. The density of states for (a) $Li_{0.33}La_{0.56}TiO_3$, (b) $Li_{0.167}La_{0.61}TiO_3$, and (c) Ti- and O-deficient $Li_{0.33}La_{0.56}TiO_3$.

Supplementary tables

Table S1: ICP-OES analysis of Li, La, and Ti in LLTO samples after different thermal treatments.

	Atomic percent (at. %) ± standard deviations		
	Li	La	Ti
LLTO powder after calcination at 800 °C, 10h	0.151 ± 0.011	0.315 ± 0.007	0.458 ± 0.011
Calcined LLTO powder after sintering at 1250 °C, 12 h	0.132 ± 0.017	0.318 ± 0.025	0.461 ± 0.030
Calcined LLTO powder after sintering at 1350 °C, 12 h	0.038 ± 0.004	0.305 ± 0.024	0.425 ± 0.037

Experimental procedure of ICP-OES

A Varian 715-ES ICP optical emission spectrometer was used to perform elemental analysis. Before analysis, 15 mg of each sample was weighed into a 50 ml glass, and 10 ml of HCl was added. The glass was covered and the contents were heated to boiling. The contents were then cooled and filtered through blue ribbon filter paper into a 50 ml centrifuge tube. The filter paper was washed thoroughly, and the filtered content was diluted to a final volume of 50 ml with MQ water (1st series of samples). The filter paper was transferred to a platinum crucible and incinerated. To the "incinerated" sample in the platinum crucible, 2 g of $KHSO_4$ was added, and it was heated until complete fusion occurred. Then, 10 ml of HCl was added to the melt, and the crucible was heated until all the fusion was dissolved. The solution was transferred to 50 ml centrifuge tubes, washed thoroughly, and diluted to a final volume of 50 ml with MQ water (2nd series of samples). Both sets of samples in the centrifuge tubes were appropriately diluted for analysis.

Table S2. Absolute intensities of the atomic columns (La, Ti-C, and Ti-E) in simulations of the $Li_2La_2Ti_3O_{10}$ phase at different thicknesses and experimental images in Figures 2c and 2d along (a) [100] and (b) [110] zone axes. Intensity ratios $I_{Ti\text{-}C}$:I_{La} and $I_{Ti\text{-}E}$:I_{La} are calculated for comparison between the experimental and simulated images.

(a) [100]		Absolute intensity ± relative standard deviations (%)			Intensity ratios (%)	
		I_{La}	$I_{Ti\text{-}C}$	$I_{Ti\text{-}E}$	$I_{Ti\text{-}C}$:I_{La}	$I_{Ti\text{-}E}$:I_{La}
Simulations	9.4 nm	58.6 ± 0.5	18.7 ± 0.5	15.8 ± 0.4	32.0	27.0
	20.9 nm	74.6 ± 0.4	32.9 ± 0.4	27.9 ± 0.3	44.1	37.5
	30.5 nm	87.1 ± 0.5	43.7 ± 0.6	37.7 ± 0.3	50.2	43.2
	40.1 nm	95.7 ± 1.1	50.5 ± 1.0	44.1 ± 1.4	52.8	46.1
	51.1 nm	111.2 ± 0.2	61.3 ± 0.3	54.0 ± 0.2	55.1	48.6
Exp. (Fig. 2c)		636 ± 3	339 ± 3	292 ± 6	45.9	53.3

(b) [110]		Absolute intensity ± relative standard deviations (%)			Intensity ratios (%)	
		I_{La}	$I_{Ti\text{-}C}$	$I_{Ti\text{-}E}$	$I_{Ti\text{-}C}$:I_{La}	$I_{Ti\text{-}E}$:I_{La}
Simulations	10.6 nm	15.7 ± 0.7	4.2 ± 1.1	3.6 ± 0.9	27.0	23.0
	21.7 nm	20.6 ± 0.6	9.0 ± 0.4	7.2 ± 0.4	43.6	34.8
	29.6 nm	23.4 ± 0.5	11.7 ± 0.4	9.4 ± 0.4	50.2	40.4
	38.8 nm	26.1 ± 0.4	14.3 ± 0.4	11.5 ± 0.4	55.0	44.2
Exp. (Fig. 2d)		802 ± 6	380 ± 6	323 ± 8	47.4	40.3

Table S3. Average absolute intensities with relative deviations (in %) of the atomic layers comprising a RP-type defect in the predicted structural models with thickness (a) 30 nm, (b) 20 nm, and (c) 15 nm along [100] zone axis and experimental HAADF-STEM image of the non-periodic sequence (Figure 3b). Intensity ratios $I_{La\text{-}C}:I_{La\text{-}E}$, $I_{Li}:I_{La\text{-}E}$, $I_{Ti\text{-}E}:I_{La\text{-}E}$, and $I_{Ti\text{-}C}:I_{La\text{-}E}$ are calculated for comparison of the experimental values with simulations.

(a) 30 nm		Absolute intensities ± relative std. deviation in %					Intensity ratios in %				
		$I_{La\text{-}E}$	$I_{La\text{-}C}$	$I_{Li\text{-}La}$	$I_{Ti\text{-}E}$	$I_{Ti\text{-}C}$	$I_{La\text{-}C}:I_{La\text{-}E}$	$I_{Li}:I_{La\text{-}E}$	$I_{Ti\text{-}E}:I_{La\text{-}E}$	$I_{Ti\text{-}C}:I_{La\text{-}E}$	
							Absolute difference to reference (experimental)				Average absolute difference
Model	Only shift	21.9 ± 0.7	14.8 ± 6.2	2.6 ± 8.1	12.0 ± 0.7	12.2 ± 0.7	67.6	11.7	54.9	55.6	
							4.7	13.5	14.1	7.5	9.9
	10La-10Ti	21.4 ± 4.6	15.6 ± 6.6	6.8 ± 15.9	11.8 ± 4.3	12.9 ± 1.0	73.2	31.7	55.4	60.4	
							0.9	6.5	14.6	12.2	8.6
	20La-10Ti	19.2 ± 5.2	15.6 ± 6.2	10.6 ± 14.2	12.3 ± 3.8	12.9 ± 1.0	81.3	55.0	63.8	67.2	
							9.0	29.8	23.0	19.0	20.2
	15La-15Ti	20.7 ± 6.5	15.6 ± 6.6	8.4 ± 11.9	11.5 ± 5.9	13.0 ± 1.1	75.5	41.0	55.3	62.5	
							3.2	15.9	14.6	14.3	12.0
	10La-20Ti	20.9 ± 2.7	14.8 ± 6.4	7.1 ± 21.0	10.4 ± 4.7	12.2 ± 1.0	70.8	34.0	49.8	58.6	
							1.5	8.9	9.0	10.4	7.4
	20La-20Ti	19.7 ± 8.3	15.6 ± 6.6	11.6 ± 14.5	11.3 ± 4.5	12.9 ± 1.2	79.4	59.0	57.3	65.6	
							7.1	33.9	16.6	17.4	18.7
Exp. (Fig. 3b)		4769 ± 6	3448 ± 10	1200 ± 12	1945 ± 6	2298 ± 5	72.3	25.2	40.8	48.2	Reference

(b) 20 nm		Absolute intensities ± relative std. deviation in %					Intensity ratios in %				
		I_{La-E}	I_{La-C}	I_{Li-La}	I_{Ti-E}	I_{Ti-C}	I_{La-C}:I_{La-E}	I_{Li}:I_{La-E}	I_{Ti-E}:I_{La-E}	I_{Ti-C}:I_{La-E}	
							Absolute difference to reference (experimental)				Average absolute difference
Model	Only shift	18.7 ± 0.8	12.7 ± 7.2	1.8 ± 8.0	8.9 ± 0.8	9.3 ± 0.8	68.0	9.4	47.6	49.6	
							4.3	15.8	6.8	1.5	7.1
	10La-10Ti	18.1 ± 5.5	13.4 ±7.7	4.9 ± 26.3	8.6 ± 4.4	9.8 ± 1.0	74.3	27.1	47.7	54.1	
							2.0	1.9	7.0	5.9	4.2
	20La-10Ti	16.3 ± 6.1	13.4 ± 7.2	8.2 ± 20.0	9.0 ± 4.5	9.8 ± 1.0	82.2	50.4	55.0	59.9	
							9.9	25.2	14.2	11.7	15.3
	15La-15Ti	17.6 ± 6.2	13.4 ± 7.7	5.8 ± 19.9	8.3 ± 6.7	9.8 ± 1.6	76.0	32.7	47.0	55.8	
							3.7	7.5	6.2	7.6	6.3
	10La-20Ti	17.8 ± 4.1	12.7 ± 7.4	5.0 ± 25.4	7.6 ± 6.0	9.3 ± 0.9	71.5	28.3	42.5	52.4	
							0.8	3.2	1.7	4.2	2.5
	20La-20Ti	16.6 ± 9.1	13.4 ± 7.7	8.8 ± 18.3	8.2 ± 7.0	9.8 ± 1.2	80.8	53.2	49.4	59.1	
							8.5	28.1	8.6	10.9	14.0
Exp. (Fig. 3b)		4769 ± 6	3448 ± 10	1200 ± 12	1945 ± 6	2298 ± 5	72.3	25.2	40.8	48.2	Reference

(c) 15 nm		Absolute intensities ± relative std. deviation in %					Intensity ratios in %				
		$I_{La\text{-}E}$	$I_{La\text{-}C}$	$I_{Li\text{-}La}$	$I_{Ti\text{-}E}$	$I_{Ti\text{-}C}$	$I_{La\text{-}C}:I_{La\text{-}E}$	$I_{Li}:I_{La\text{-}E}$	$I_{Ti\text{-}E}:I_{La\text{-}E}$	$I_{Ti\text{-}C}:I_{La\text{-}E}$	
							Absolute difference to reference (experimental)				Average absolute difference
Model	Only shift	16.6 ± 0.8	11.5 ±9.0	1.3 ± 5.8	6.8 ± 0.8	7.4 ± 0.7	69.2	7.6	40.9	44.5	
							3.1	17.6	0.1	3.7	6.1
	10La-10Ti	15.3 ± 6.8	11.5 ± 9.5	3.4 ± 39.2	6.2 ± 5.1	7.4 ± 1.1	75.3	22.3	40.4	48.3	
							3.0	2.9	0.4	0.1	1.6
	20La-10Ti	13.7 ± 6.8	11.5± 9.0	6.1 ± 26.8	6.4 ± 5.9	7.4 ± 0.9	83.8	44.2	46.5	53.8	
							11.5	19.1	5.7	5.6	10.5
	15La-15Ti	14.9 ± 6.8	11.5± 9.6	4.0 ± 22.5	5.8 ± 7.1	7.4 ± 1.4	77.1	27.0	38.7	49.6	
							4.8	1.8	2.1	1.4	2.5
	10La-20Ti	15.9 ± 4.8	11.5± 9.3	3.8 ± 32.7	5.6 ± 6.7	7.4 ± 1.0	72.4	23.9	35.4	46.6	
							0.1	1.3	5.4	1.6	2.1
	20La-20Ti	14.0 ± 9.9	11.5± 9.5	6.6 ± 27.8	5.9 ± 8.5	7.4 ± 1.2	82.1	47.3	42.0	52.8	
							9.8	22.1	1.2	4.6	9.4
Exp. (Fig. 3b)		4769 ± 6	3448 ± 10	1200 ± 12	1945 ± 6	2298 ± 5	72.3	25.2	40.8	48.2	Reference
